# TBK-binding protein 1 regulates IL-15-induced autophagy and NKT cell survival

**DOI:** 10.1038/s41467-018-05097-5

**Published:** 2018-07-18

**Authors:** Lele Zhu, Xiaoping Xie, Lingyun Zhang, Hui Wang, Zuliang Jie, Xiaofei Zhou, Jianhong Shi, Shuli Zhao, Boxiang Zhang, Xuhong Cheng, Shao-Cong Sun

**Affiliations:** 1 Department of Immunology, The University of Texas MD Anderson Cancer Center, 7455 Fannin Street, Box 902, Houston, TX 77030 USA; 2 Center for Reproductive Medicine, Henan Key Laboratory of Reproduction and Genetics, The First Affiliated Hospital of Zhengzhou University, Zhengzhou, 450000, China; 3 Department of Pathogenic Biology and Immunology, Xuzhou Medical University, 209 Tongshan Road, Xuzhou, Jiangsu, 221004 China; 4 Central Laboratory, Affiliated Hospital of Hebei University, 212 Yuhua East Road, 07100 Baoding, China; 5 General Clinical Research Center, Nanjing First hospital, Nanjing Medical University, Nanjing, Jiangsu, 210012 China; 6 Department Two of Thoracic Surgery, The First Affiliated Hospital of Xi’an Jiaotong University, Xi’an, Shaanxi, 710061 China; 7 The University of Texas Graduate School of Biomedical Sciences, Houston, TX 77030 USA

## Abstract

The cytokine IL-15 mediates development and survival of immune cells, including natural killer T (NKT) cells, but the underlying mechanism of IL-15 function is incompletely understood. Here we show that IL-15 induces autophagy in NKT cells with a mechanism that involves a crucial signaling component, TBK-binding protein 1 (Tbkbp1). Tbkbp1 facilitates activation of the autophagy-initiating kinase Ulk1 through antagonizing the inhibitory action of mTORC1. This antagonization involves the recruitment of an mTORC1-opposing phosphatase to Ulk1. Tbkbp1 deficiency attenuates IL-15-stimulated NKT cell autophagy, and is associated with mitochondrial dysfunction, aberrant ROS production, defective Bcl2 expression and reduced NKT cell survival. Consequently, Tbkbp1-deficient mice have profound deficiency in NKT cells, especially IFN-γ-producing NKT1. We further show that Tbkbp1 regulates IL-15-stimulated autophagy and survival of NK cells. These findings suggest a mechanism of autophagy induction by IL-15, and establish Tbkbp1 as a regulator of NKT cell development and survival.

## Abstract (web summary)

Interleukin-15 (IL-15) regulates the homeostasis of many immune cell types, including natural killer T (NKT) cells, but the underlying mechanism is not completely clear. Here the authors analyse *Tbkbp1*-deficient mice and show that IL-15 induces Tbkbp1-dependent autophagy to modulate NKT survival.

## Introduction

Autophagy is a multi-step cellular process that delivers unused proteins and damaged organelles to the lysosome for breakdown, thereby promoting cell survival under extreme conditions such as nutrient deprivation^1^. The initiation of autophagy involves formation of a protein complex, composed of UNC51-like kinase (Ulk1 or Ulk2), the scaffold protein FIP200 (also called RB1CC1), autophagy-related (ATG) 13 and ATG101^2^. Upon activation, Ulk1/2 phosphorylates downstream targets, including BECLIN1 and VPS34, involved in phagophore formation. Subsequent events involve lipidation of microtubule-associated protein 1 light chain 3 (LC3) to convert it from a cytosolic form (LC3-I) to a lapidated form (LC3-II) that is recruited to autophagosomal membranes, where it mediates cargo recruitment and autophagosome completion. Eventually, autophagosomes fuse with lysosomes to form autolysosomes, in which cargos are degraded by lysosomal hydrolases^2^. A key step in autophagy induction is activation of Ulk1, which is reciprocally regulated by mammalian target of rapamycin (mTOR) complex 1 (mTORC1) and AMP-activated kinase alpha (AMPKa)^2,3–5^. Under nutrient-competent conditions, mTORC1 inhibits autophagy through phosphorylating Ulk1 at serine 757, which prevents Ulk1 binding and activation by AMPKa; nutrient deprivation inactivates mTORC1, allowing the activated AMPKa to phosphorylate Ulk1 at S555 and other activation sites for autophagy initiation^4^. Recent studies demonstrate that autophagy also plays a crucial role in physiological processes, including immune cell development and homeostasis^6–10^. However, it is unclear how autophagy is induced along with the physiological processes of immune cell development and homeostasis and how autophagy regulates immune cell survival.

Natural killer T (NKT) cells are a subset of innate-like T cells responding to lipid antigens and regulating diverse aspects of immune and autoimmune responses^11,12^. The development of NKT cells occurs in the thymus, where they originate from CD4^+^CD8^+^ double-positive (DP), and possibly also CD4^–^CD8^–^ double-negative (DN), thymocytes with a rearranged semi-invariant T-cell receptor (TCR)^11,13^. In contrast to the development of conventional T cells, which relies on self-peptide antigens presented on classical MHC molecules for positive selection, the development of NKT cells requires self-lipid antigens presented by CD1d expressed on DP thymocytes^11^. Following positive selection, immature NKT cells go through sequential stages of maturation that can be defined based on surface expression of CD44 and NK1.1 markers, including stage 1 (CD44^–^NK1.1^–^), stage 2 (CD44^+^NK1.1^–^), and stage 3 (CD44^+^NK1.1^+^). Recent studies suggest that mature NKT cells can be classified into three sublineages, NKT1, NKT2, and NKT17, characterized by expression of the transcription factors T-bet, GATA3, and RORγt, respectively, and production of the cytokines IFNγ, IL-4, and IL-17, respectively^14^. In fact, the previously defined stage 2 cells include not only immature NKT1 cells but also mature NKT2 and NKT17 cells that display CD44^+^NK1.1^–^ surface markers^15^. The expression of IL-17 receptor beta (IL-17RB) on NKT2 and NKT17 cells, but not on NKT1 sublineage cells, provides a means of lineage distinction^15^.

The requirement of autophagy in NKT cell survival and maturation has been demonstrated using mouse models carrying deficiencies in major components of the autophagy pathway^8,9^. Deletion of ATG5 or ATG7 results in severe loss of NKT cells, with predominant effect on the mature NKT cells producing interferon gamma (IFNγ)^8,9^. However, how autophagy is induced and regulated under the physiological conditions of NKT cell development and homeostasis has been undefined. Common gamma chain (γc) family of cytokines, particularly IL-15, are crucial for the survival and maturation of iNKT cells^16–18^. IL-15 deficiency predominantly impairs the homeostasis and survival of IFNγ-producing stage 3 NKT (NKT1) cells^16,18^, which is consistent with the high level expression of the beta chain of IL-2 and IL-15 receptors (IL-2/IL-15R) on these cells^19,20^. On the other hand, the survival of RORγt^+^ NKT17 cells is independent of IL-15 but relies on IL-7^21^. However, the molecular mechanism underlying the survival function of IL-15 is incompletely understood.

In the present study, we show that IL-15 stimulates an autophagy pathway that is crucial for the survival of NKT cells. In contrast to nutrient deprivation, which inactivates mTORC1 and activates AMPKa^4^, IL-15 activates both AMPKa and mTORC1 and requires a signaling factor, TBK-binding protein 1 (Tbkbp1), for Ulk1 activation. We provide genetic evidence that *Tbkbp1* deficiency attenuates IL-15-stimulated NKT cell autophagy, causing mitochondrial dysfunction and aberrant ROS production, as well as impaired survival gene expression and apoptosis of the Tbkbp1-deficient NKT cells. Consequently, the Tbkbp1-deficient mice have a profoundly reduced number of NKT cells, predominantly the IFNγ-producing NKT1 cells. We further show that Tbkbp1 is also required for IL-15-induced autophagy and survival of NK cells. These findings provide insight into the mechanisms underlying autophagy induction and function in the physiological process of immune cell development and establish Tbkbp1 as a regulator of NK and NKT cell survival.

## Results

### Tbkbp1 deficiency reduces IFNγ-producing NKT1 cells

**Fig. 1:**
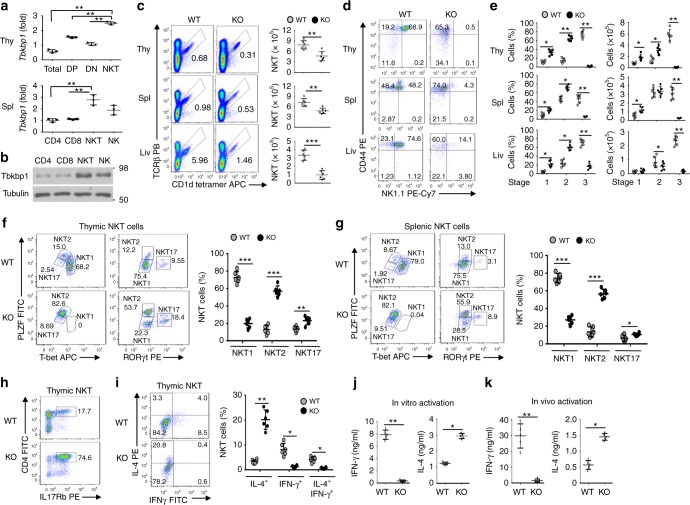
Tbkbp1 is abundantly expressed in NKT cells and has a cell-intrinsic role in regulating NKT cell development. **a** qRT-PCR analyses of *Tbkbp1* mRNA in total, CD4^+^CD8^+^ double-positive (DP), CD4^–^CD8^–^ double-negative (DN), and NKT thymic populations (upper) or splenic CD4^+^ and CD8^+^ T cells, NKT cells, and NK cells (lower). **b** IB analysis of Tbkbp1 and loading control Tubulin in the indicated splenic cell populations. **c** Flow cytometric analysis of NKT cell frequency and absolute numbers in the thymus (Thy), spleen (Spl) and liver (Liv) of age-matched WT and *Tbkbp1*-KO (KO) mice, presented as representative plots (left) and summary graphs (right). *n* = 6 per genotype. **d**, **e** Flow cytometric analysis of NKT cell maturation stages (stage1: NK1.1^–^CD44^–^; stage2: NK1.1^–^CD44^+^; stage 3: NK1.1^+^CD44^+^) in the thymus, spleen and liver of WT and *Tbkbp1*-KO mice, presented as representative plots (**d**) and summary graphs (**e**). *n* = 6 per genotype. **f**, **g** Flow cytometric analysis of the indicated transcription factors in thymic (**f**) and splenic (**g**) NKT cells from WT and *Tbkbp1*-KO mice, presented as representative plots (left) and summary graphs based on PLZF/RORγt flow values (right). *n* = 6 per genotype. **h** Flow cytometric analysis of thymic NKT cells based on IL-17Rb expression. **i** Flow cytometric analysis of IFNγ and IL-4 expression in WT and *Tbkbp1*-KO thymic NKT cells after treatment for 4 h with PMA and Ionomycin in the presence of monensin, presented as representative plots and summary graphs (*n* = 6 per genotype). **j** ELISA of IFN-γ and IL-4 in the supernatant of WT and *Tbkbp1-KO* thymic NKT cells after 48 h of in vitro stimulation with α-Galcer and antigen-presenting cells (WT BMDCs). **k** ELISA of IFNγ and IL-4 in the serum of WT and *Tbkbp1-*KO mice injected with α-Galcer (4 μg) for 6 h (*n* = 5 per genotype). Data are representative of three independent experiments, and bar graphs are presented as mean ± s.d. values. *P<0.05; **P<0.01; ***P<0.001. One-way ANOVA (**a**), Mann–Whitney test (**e**, **i**), or Student’s *t*-test (**c**, **f**, **g**, **j**, **k**)

Tbkbp1, also called SINTBAB, was identified as a protein physically interacting with the protein kinase TBK1, although its physiological function has not been defined^22^. A recent study has identified Tbkbp1 as a gene highly expressed in mature NKT cells^19^. Consistently, we found that thymic and splenic NKT cells, as well as NK cells, expressed much higher levels of Tbkbp1 than DP and DN thymocytes and conventional CD4^+^ and CD8^+^ T cells (Fig. 1a, b). Similar results were obtained with human NKT cells (Supplementary Fig. 1a–c). To examine the function of Tbkbp1, we generated Tbkbp1 germ-line knockout (*Tbkbp1*-KO) and wildtype (WT) control mice (Supplementary Fig. 1d–g). The *Tbkbp1*-KO mice had normal frequencies of thymocyte and peripheral T cell populations, except for a moderate increase in CD8^+^ single-positive (SP) thymocytes and CD8^+^ splenic T cells (Supplementary Fig. 1h–j). However, compared to the WT control mice, the *Tbkbp1*-KO mice had a significant reduction in the frequency and absolute numbers of thymic, splenic, and liver NKT cells, detected based on their binding to a glycolipid antigen (PBS57)-loaded CD1d-tetramer but not to an unloaded CD1d-tetramer (Fig. 1c and Supplementary Fig. 2a). Further analysis based on the CD44 and NK1.1 surface markers revealed that the Tbkbp1 deficiency caused a predominant loss of the stage 3 NKT cells (CD44^+^NK1.1^+^) and a relative increase in the stage 1 (CD44^–^NK1.1^–^) and stage 2 (CD44^+^NK1.1^–^) NKT cells (Fig. 1d, e).

Recent studies suggest that mature NKT cells include three distinct sublineages, NKT1, NKT2, and NKT17, with the latter two sublineages being included in the previously defined stage 2 NKT cells^14,15^. So, the question was raised as to whether Tbkbp1 regulated the development or survival of the different sublineages of NKT cells. We thus analyzed the NKT sublineages by flow cytometry based on their lineage transcription factors as well as another NKT-associated transcription factor, PLZF, as previously described^14^. The Tbkbp1 deficiency resulted in a severe loss of the PLZF^low^T-bet^+^ NKT1 subset, with a concomitant increase in the frequency of PLZF^hi^T-bet^–^ NKT2 cells, in the thymus and spleen (Fig. 1f, g). The *Tbkbp1*-KO mice also had a moderate increase in the frequency of PLZF^low^RORγt^+^ NKT17 cells (Fig. 1f, g). These results suggest that Tbkbp1 is selectively required for the development or survival of NKT1 cells.

To further determine the role of Tbkbp1 in regulating NKT subsets, we analyzed the NKT cell sublineages based on expression of IL-17Rb, a marker expressed on NKT2 and NKT17 cells but not on NKT1 cells^15,20^. We also included CD4 as a marker to further distinguish NKT2 (CD4^+^) from NKT17 (CD4^–^) cells (NKT1 cells include both CD4^+^ and CD4^–^ populations)^14^. Consistent with the loss of NKT1 cells (IL-17Rb^–^), the *Tbkbp1*-KO mice had a drastic increase in the frequency of IL-17Rb^+^ NKT cells, most strikingly the CD4^+^IL-17Rb^+^ NKT2 cells (Fig. 1h). This result further confirmed the selective loss of NKT1 and relative accumulation of NKT2 cells in *Tbkbp1*-KO mice. We next examined the possible role of Tbkbp1 in NKT sublineage commitment by gating on immature stage 1 (CD44^–^NK1.1^–^) NKT cells (Supplementary Fig. 2b) known to be the branching point of NKT1 cells from NKT2 and NKT17 cells^15^. Interestingly, the WT and *Tbkbp1*-KO mice had comparable frequencies of IL-17Rb^+^ cells during the CD44^–^NK1.1^–^ immature stage but display a striking difference in the later stage (CD44^+^NK1.1^–^), suggesting that Tbkbp1 was dispensable for NKT sublineage commitment but required for NKT1 maturation or survival (Supplementary Fig. 2c). Taken together, these results suggest that the Tbkbp1 deficiency causes a predominant loss of NKT1 cells and relative accumulation of NKT2 and NKT17 cells.

A major functional characteristic of NKT2 cells is abundant expression of IL-4 as opposed to the predominant production of IFNγ by NKT1 cells^14^. Consistent with their severe loss of NKT1 and relative increase in NKT2 subpopulation, the Tbkbp1-deficient NKT cells were largely devoid of IFNγ-producing NKT cells with a relative increase in IL-4-producing NKT cells (Fig. 1i). ELISA also revealed that *Tbkbp1*-KO NKT cells produced much less IFNγ and profoundly more IL-4 than WT NKT cells upon in vitro activation by anti-CD3 plus anti-CD28 or in vivo activation by α-GalCer (Fig. 1j, k). Thus, Tbkbp1 deficiency causes a severe loss of IFNγ-producing NKT cells and a relative accumulation of IL-4-producing NKT cells.

### Tbkbp1 is a cell-intrinsic regulator of NKT cell development

**Fig. 2:**
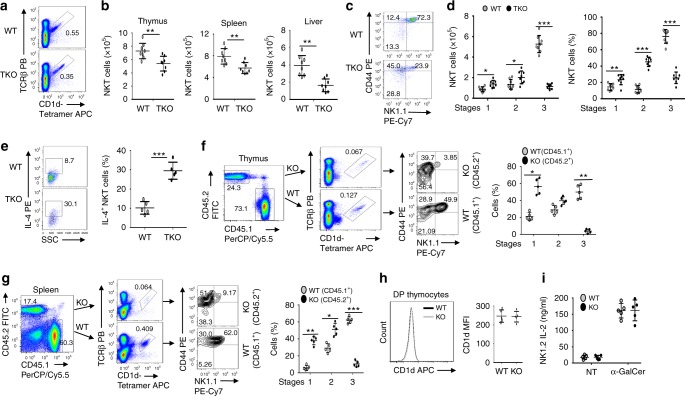
Tbkbp1 has a cell-intrinsic role in regulating NKT cell development. **a**, **b** Flow cytometric analysis of NKT cells in the thymus, spleen, and liver from 6-week-old WT and *Tbkbp1*-TKO (TKO) mice, presented as a representative FACS plot of thymic NKT cells (**a**) and summary graphs of the indicated NKT cells (each circle represents a mouse) (**b**). **c**, **d** Flow cytometric analysis of thymic NKT cell maturation stages (stage 1: CD44^–^NK1.1^–^; stage 2: CD44^+^NK1.1^–^; stage 3: CD44^+^NK1.1^+^), presented as a representative plot (**c**) and summary graphs (**d**). **e** Flow cytometric analysis of IL-4 expression in WT and *Tbkbp1*-TKO thymic NKT cells after treatment for 4 h with PMA and ionomycin in the presence of monensin, presented as a representative plot and summary graph (*n* = 5 per genotype). **f**, **g** Flow cytometric analysis of NKT cells and their maturation stages in the thymus (**f**) and spleen (**g**) of *Rag1*-KO recipient mice adoptively transferred (for 6 week) with a mixture of BM cells derived from WT B6.SJL mice (CD45.1^+^) and *Tbkbp1*-KO mice (CD45.2^+^), gating on CD45.1^+^ or CD45.2^+^ cells and presented as representative FACS plots and summary graphs (*n* = 5 chimeric mice). **h** Flow cytometric analysis of cell surface expression of CD1d in DP thymocytes from WT or *Tbkbp1-KO* mice, presented as a representative FACS plot and summary graph (*n* = 5 mice per genotype). **i** ELISA of IL-2 produced by NKT hybridoma cells cocultured for 24 h with total thymocytes from WT or *Tbkbp1*-KO mice in the absence or presence of α-GalCer. Data are representative of at least three independent experiments, and bar graphs are presented as mean ± s.d. values. *P < 0.05; **P < 0.01; ***P < 0.001. Student’s *t*-test (**b**, **d**–**i**)

To examine the cellular mechanism of Tbkbp1 function, we generated T cell-conditional *Tbkbp1* KO (*Tbkbp1*-TKO) mice (Supplementary Fig. 1d–g). As seen with the whole-body *Tbkbp1*-KO mice, the *Tbkbp1*-TKO mice had reduced frequency and number of NKT cells in both the thymus and peripheral organs (Fig. 2a, b). Moreover, the loss of NKT cells in the *Tbkbp1*-TKO mice was predominantly in the CD44^+^NK1.1^+^ (previously known as stage 3) population, which was coupled with a relative increase in the CD44^–^NK1.1^–^ (stage 1) and CD44^+^NK1.1^–^ (stage 2) cells producing IL-4 (Fig. 2c–e). These results suggested a T cell-specific function of Tbkbp1 in regulating NKT cell development.

Since thymocytes serve as both NKT precursors and antigen-presenting cells that support NKT cell development^11^, the *Tbkbp1*-TKO mice could not determine whether Tbkbp1 had a cell-intrinsic or cell-extrinsic function in NKT cell regulation. To solve this problem, we carried out mixed bone marrow (BM) adoptive transfer studies by transferring *Rag1*-KO mice with a mixture of WT (CD45.1^+^) and *Tbkbp1*-KO (CD45.2^+^) BM cells. The chimeric mice had a drastically reduced frequency of stage 3 NKT cells derived from the CD45.2^+^ *Tbkbp1*-KO BM, but this phenotype was not detected in NKT cells derived from the CD451.1^+^ WT BM (Fig. 2f, g). In agreement with these results, the Tbkbp1 deficiency did not influence the antigen-presentation function of DP thymocytes, including expression of the non-classical MHCI molecule CD1d (Fig. 2h) and ability to mediate antigen-specific NKT cell activation (Fig. 2i). Collectively, these results demonstrate a cell-intrinsic role for Tbkbp1 in regulating NKT cell development and maturation.

### Tbkbp1 deficiency increases memory-like CD8^+^ T cells

**Fig. 3:**
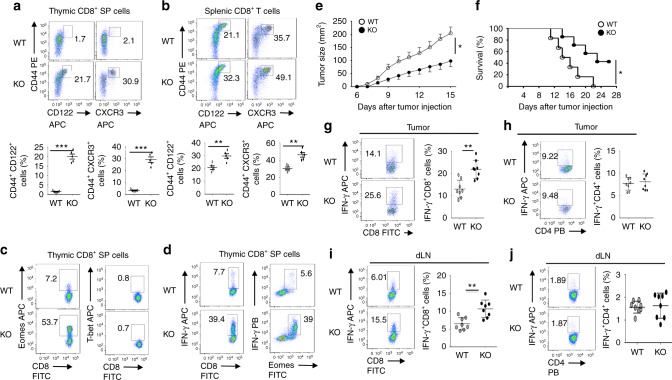
Tbkbp1 deficiency increases the frequency of memory-like CD8^+^ T cells and antitumor immunity. **a**, **b** Flow cytometric analysis of memory-like CD8^+^ T cells based on expression of CD44, CD122, and CXCR3 surface markers in the CD8^+^ single positive (SP) thymocytes (**a**) and spleen CD8^+^ T cells (**b**) derived from 6-week-old WT and *Tbkbp1-KO* (KO) mice, presented as representative FACS plots (upper) and summary graphs (lower, each circle represents a mouse). **c**, **d** ICS analysis of Emoes^+^ and T-bet^+^ cells (**c**) and IFNγ^+^ and IFNγ^+^Eomes^+^ cells (**d**) in CD8^+^ single positive (SP) thymocytes. **e**, **f** Tumor growth (**e**) and survival (**f**) curves of WT and *Tbkbp1-KO* (KO) mice injected s.c. with B16-OVA melanoma cells. Lethality was defined as tumor size reaching to 225 mm^2^. *n* = 8 per genotype group. **g**–**j** Flow cytometric analysis of the frequency of IFNγ-producing CD8^+^ (**g**, **i**) and CD4^+^ (**h**, **j**) T cells in the tumors (**g**, **h**) or draining lymph node (**i**, **j**) of WT and *Tbkbp1-KO* mice injected s.c. with B16-OVA melanoma cells (day 15 after injection), presented as representative plots (left) and summary graphs (right). Data are representative of at least three independent experiments, and bar graphs are presented as mean ± s.d. values. *P < 0.05; **P < 0.01; ***P < 0.001. Student’s *t*-test (**b**, **g**, **i**), Mann–Whitney test (**a**, **h**, **j**), Two-way ANOVA (**e**), log-rank Mantel-Cox test (**f**)

The NK1.1^–^ (predominantly NKT2) cells mediate steady-state production of IL-4, which promotes development of memory-like CD8^+^ T cells^14,23,24^. Consistent with their relative increase inIL-4-producingNKT cells, the *Tbkbp1-KO* mice had a drastic increase in the frequency of thymic and splenic memory-like CD8^+^ T cells, characterized by the CD44^hi^CD122^+^ and CD44^hi^CXCR3^+^ surface markers (Fig. 3a, b). The *Tbkbp1-KO* CD8^+^ SP thymocytes also expressed high levels of the transcription factor Eomes but not T-bet (Fig. 3c), a hallmark of IL-4-induced memory-like CD8^+^ T cells^24^. Moreover, these cells were capable of rapid production of the effector cytokine IFNγ upon in vitro stimulation (Fig. 3d). Similar results were obtained with the *Tbkbp1*-TKO mice (Supplementary Fig. 3a, b).

Because the *Tbkbp1*-TKO mice harbor Tbkbp1 deficiencies in both conventional T cells and NKT cells, we next performed mixed bone-marrow adoptive transfer studies to determine whether Tbkbp1 functioned cell-intrinsically in CD8^+^ T cells or might function in supporting cells to regulate memory-like CD8^+^ T cell generation. In the mixed BM chimeric mice, the CD8^+^ T cells derived from the WT and *Tbkbp1*-KO BM had similar frequencies of memory-like population (Supplementary Fig. 3c), suggesting a cell-extrinsic function of Tbkbp1 in regulating memory-like CD8^+^ T cell generation. Steady-state IL-4 production in naïve mice is known to occur predominantly in NK1.1^–^ NKT cells^23^, which is required for memory-like CD8 T cell generation^14^. Since Tbkbp1 deficiency caused relative increase in IL-4-producing NKT cells, we examined the involvement of IL-4 by crossing *Tbkbp1*-KO mice with *Il4*-KO mice. IL-4 deficiency had no obvious effect on NKT cell development, but blocked the IL-4 production in *Tbkbp1-KO* NKT cells (Supplementary Fig. 3d). Interestingly, deletion of IL-4 completely reversed the CD8^+^ memory-like CD8^+^ T cells (Supplementary Fig. 3e). These results suggest the enhanced memory-like CD8^+^ T cell generation in *Tbkbp1*-KO mice is indirectly caused by the impaired NKT cell maturation.

To assess the in vivo role of Tbkbp1 in regulating CD8^+^ T cell responses, we employed a tumor immunity model involving inoculation of B16 murine melanoma cells expressing a surrogate antigen, chicken ovalbumin (OVA), to *Tbkbp1-KO* and WT mice. Compared to WT mice, the *Tbkbp1-KO* mice had reduced tumor growth rate and improved survival rate (Fig. 3e, f), coupled with increased frequencies of IFNγ-producing CD8^+^ effector T cells in the tumor and draining lymph node (Fig. 3g, i). On the other hand, the frequency of CD4^+^ effector T cells was comparable between the WT and *Tbkbp1-KO* mice, suggesting that the elevated antitumor immunity in *Tbkbp1-KO* mice might be due to the increase in memory-like CD8^+^ T cells. In support of this idea, deletion of IL-4 in *Tbkbp1-KO* mice, which blocked hyper-production of memory-like CD8^+^ T cells (Supplementary Fig. 3e), abrogated their ability to mediate stronger tumor rejection and CD8^+^ effector T cell responses (Supplementary Fig. 4a–c). Furthermore, depletion of CD8^+^ T cells using an anti-CD8 neutralizing antibody (Supplementary Fig. 4f) markedly attenuated the antitumor immunity of the *Tbkbp1-KO* mice and erased the difference between the *Tbkbp1*-KO and WT control mice (Supplementary Fig. 4g). These results suggest that Tbkbp1 regulates IL-4-dependent generation of memory-like CD8^+^ T cells with antitumor function.

### Tbkbp1 is required for IL-15-stimulated NKT cell survival

**Fig. 4:**
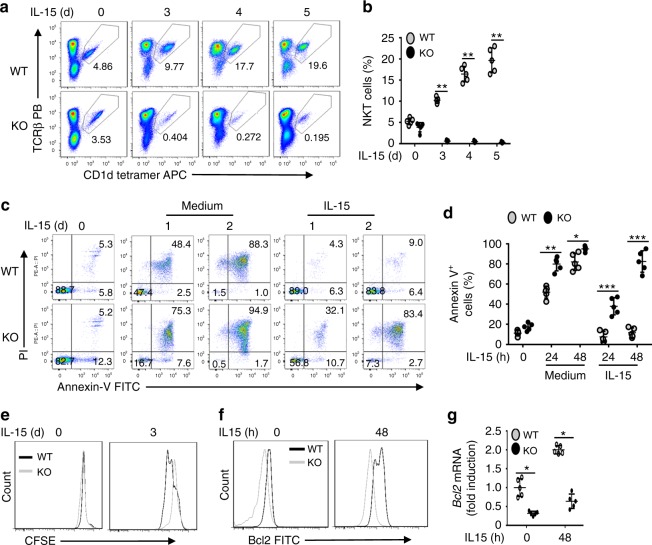
Tbkbp1 mediates IL-15-stimulated NKT cell survival. **a**, **b** Flow cytometric analysis of NKT cell frequency in NKT-enriched WT and *Tbkbp1*-KO thymocytes (CD8^+^ cells depleted by microbeads) after in vitro incubation with IL-15 for the indicated time periods. Data are presented as a representative plot (**a**) and summary graph (**b**). **c**, **d** Flow cytometry analysis of apoptotic cells (based on AnnexinV and PI staining) in enriched thymic NKT cells cultured for indicated time periods with IL-15 or medium control. Data are presented as a representative plot (**c**) and summary graph (**d**). **e** Proliferation assays (based on CFSE dilution) of WT or *Tbkbp1*-KO thymic NKT cells labeled with CFSE and cultured for the indicated time with IL-15. **f**, **g** Flow cytometric analysis of intracellular Bcl-2 level (**f**) and qRT-PCR analysis of Bcl2 mRNA (**g**) in WT or *Tbkbp1*-KO thymic NKT cells, either freshly isolated or cultured with IL-15 for 48 h, presented as a representative plot (left) and summary graph (right). All summary graphs are presented as mean ± s.d. values based on 5 WT and 5 *Tbkbp1*-KO mice. The similar data were obtained from at least three independent experiments. *P < 0.05; **P < 0.01; ***P < 0.001. Mann–Whitney test (**b**), Student’s *t*-test (**d**, **g**)

The cytokine IL-15 plays a crucial role in NKT survival and maturation^16–18^. In particular, the IL-17Rb^–^ IFNγ-producing NKT1 cells express high levels of IL-2/IL-15Rb and rely on IL-15 for survival^16,18–20^, although the survival of IL-17Rb^+^ NKT2 and NKT17 cells is independent of IL-15^20,21^. Because of the selective role of Tbkbp1 in NKT1 regulation, we examined the role of Tbkbp1 in regulating IL-15-induced NKT cell survival and proliferation. IL-15 induced time-dependent expansion of WT NKT cells, which was almost completely blocked in the *Tbkbp1*-KO NKT cells (Fig. 4a, b). Parallel apoptosis analysis revealed that in vitro cultured NKT cells were undergoing massive apoptosis (Fig. 4c, d). Importantly, the apoptosis of WT NKT cells, but not *Tbkbp1-KO* NKT cells, was efficiently protected by IL-15 (Fig. 4c, d). The *Tbkbp1-KO* NKT cells also had a moderate defect in IL-15-induced proliferation (Fig. 4e). IL-15-mediated inhibition of NKT cell apoptosis involves induction of survival factors, including Bcl-2^25^. Flow cytometry and qRT-PCR analyses of freshly isolated NKT cells revealed a reduction in Bcl-2 expression in the *Tbkbp1-KO* NKT cells, suggesting defective responses to in vivo homeostatic triggers (Fig. 4f, g). Moreover, IL-15 upregulated the expression level of Bcl-2 protein and mRNA in WT NKT cells but barely induced Bcl-2 expression in *Tbkbp1-KO* NKT cells (Fig. 4f, g). These results suggest that Tbkbp1 is a critical mediator of IL-15 induced Bcl2 expression and NKT cell survival.

Since IL-15 and IL-2 stimulate signaling via the same receptor subunits, IL-2/IL-15b and common gamma chain (γc)^26^, we wondered whether Tbkbp1 also played a role in regulating IL-2-stimulated signaling in NKT cells. Like IL-15, IL-2 prevented apoptosis of WT NKT cells (Supplementary Fig. 5a, b). Importantly, the IL-2-stimulated NKT survival was impaired in *Tbkbp1*-KO NKT cells (Supplementary Fig. 5a, b). On the other hand, Tbkbp1 was largely dispensable for the induction of NKT cell survival mediated by another γc family cytokine IL-7 (Supplementary Fig. 5a, b), even when tested with a broad range of doses (Supplementary Fig. 5c, d). Consistently, Tbkbp1 deficiency attenuated Bcl-2 expression in NKT cells stimulated by IL-2 and IL-15, but not by IL-7 (Supplementary Fig. 5e).

### Tbkbp1 regulates mitochondria ROS

**Fig. 5:**
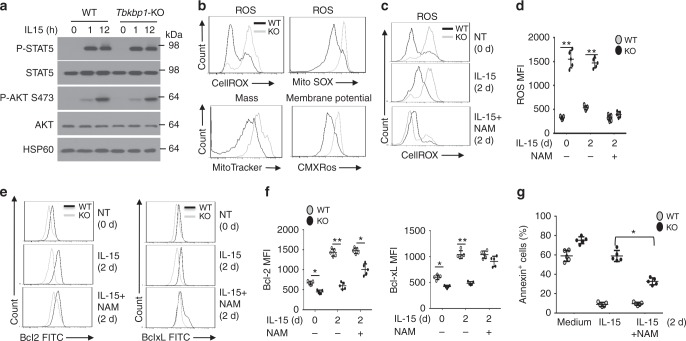
Tbkbp1-mediated NKT cell survival involves regulation of mitochondria ROS. **a** IB analyses of the indicated phosphorylated (P-) and total proteins of NKT cells stimulated with IL-15 for the indicated time periods. **b** Flow cytometric analysis of WT and *Tbkbp1*-KO thymic NKT cells that were stained with CellROX^TM^ Deep Red and MitoSOX for detection of ROS or stained with MitoTracker Green and MitoTracker Red CMXRos for detection of mitochondrial mass and membrane potential, respectively. **c**, **d** Flow cytometric analysis of ROS concentration in WT and *Tbkbp1*-KO thymic NKT cells that either untreated (0 h) or cultured for 2 days with IL-15 or IL-15 plus NAM. Data are presented as a representative plot (**c**) and summary graph (**d**). *n* = 5 per genotype group. **e**, **f** Flow cytometric analysis of intracellular Bcl-2 or Bcl-xL level in WT and *Tbkbp1*-KO thymic NKT cells that either untreated (0 h) or cultured for 2 days with IL-15 or IL-15 plus NAM. Data are presented as representative plots (**e**) and summary graphs (**f**). *n* = 5 mice per genotype group. **g** Flow cytometry quantification of AnnexinV^+^ apoptotic cells in thymic NKT cells cultured for 2 days with medium control, IL-15, or IL-15 plus NAM, presented as a summary graph. *n* = 5 mice per genotype group. Data are representative of three independent experiments, and bar graphs are presented as mean ± s.d. values. *P < 0.05; **P < 0.01. Student’s *t*-test (**d**, **f**, **g**)

We next examined the molecular mechanism by which Tbkbp1 mediates IL-15-stimulated Bcl2 expression and NKT cell survival. Surprisingly, the Tbkbp1 deficiency did not appreciably influence IL-15-stimulated phosphorylation of the transcription factor STAT5 and the survival kinase AKT (Fig. 5a), two major signaling events stimulated by IL-15^27^. These results suggested that Tbkbp1 might regulate a downstream molecular event involved in Bcl-2 induction and NKT cell survival. In this regard, reactive oxygen species (ROS) are known to suppress Bcl-2 gene expression and promote cell death^28^. Analysis of freshly isolated NKT cells revealed that while the majority of WT NKT cells had a low content of ROS, almost the entire population of Tbkbp1-deficient NKT cells had a high content of ROS, suggesting aberrant ROS production under in vivo homeostatic conditions (Fig. 5b). The increased ROS production in the *Tbkbp1-KO* NKT cells was also detected based on staining using MitoSOX Red (Fig. 5b), a dye known to detect mitochondrial superoxide anion^29^.

Mitochondria is a major source of ROS, and aberrant ROS production is associated with mitochondrial dysfunction^30^. The increased ROS production in Tbkbp1-deficient NKT cells prompted us to examine the effect of Tbkbp1 deficiency on mitochondrial function based on staining with MitoTracker Green and MitoTracker Red CMXRos, known to detect mitochondrial content and membrane potential, respectively^29^. Compared to the WT NKT cells, the Tbkbp1-deficient NKT cells displayed profoundly increased mitochondrial mass and membrane potential, indicative of mitochondrial dysfunction (Fig. 5b).

To assess the possible connection between aberrant ROS production and impaired survival of Tbkbp1-deficient NKT cells, we performed in vitro studies by employing nicotinamide (NAM), a precursor of the coenzyme NAD^+^ known to regulate mitochondrial potential and inhibit ROS production^31^. Following in vitro cultivation in the presence of IL-15, KO NKT cells still had substantially higher levels of ROS than WT NKT cells (Fig. 5c, d). As expected, the ROS level in both WT and KO NKT cells could be efficiently lowered down upon incubation with NAM (Fig. 5c). Importantly, NAM treatment largely, although not completely, rescued the defect of the Tbkbp1-deficient NKT cells in IL-15-stimulated expression of Bcl-2 and Bcl-XL (Fig. 5e, f). The NAM treatment also partially restored IL-15-induced survival of the Tbkbp1-deficient NKT cells (Fig. 5g). These results suggest that Tbkbp1 deficiency in NKT cells causes mitochondrial dysfunction and aberrant ROS production, which contributes to the defect in IL-15-induced survival gene expression and apoptosis inhibition.

### Tbkbp1 mediates IL-15-stimulated autophagy in NKT cells

**Fig. 6:**
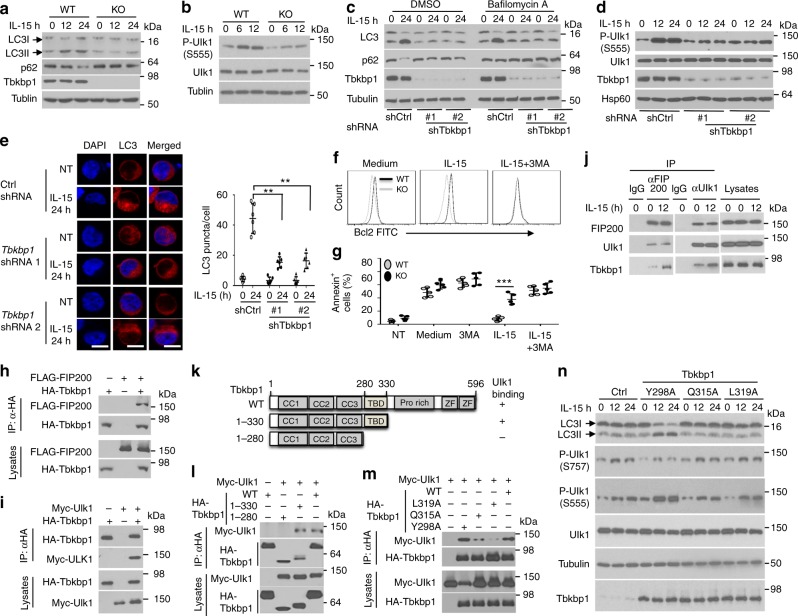
Tbkbp1 mediates IL-15-stimulated autophagy in NKT cells. **a**–**d** IB analysis of LC3 modification and p62 degradation (**a**, **c**) and Ulk1 phosphorylation (**b**, **d**) in whole-cell lysates of IL-15-stimulated WT or *Tbkbp1*-KO thymic NKT cells (**a**, **b**) or NKT hybridoma cells transduced with a non-silencing control shRNA (shCtrl) or two different *Tbkbp1*-specific shRNAs that were stimulated with IL-15 (**d**) or IL-15 together with DMSO or bafilomycin A (**c**). **e** Confocal microscopic analysis of LC3 puncta, DAPI (nuclear staining), and merged picture of untreated (NT) or IL-15-stimulated control or *Tbkbp1*-knockdown NKT hybridoma cells. Data are presented as representative images (left) and summary graph of quantified LC3 puncta (right). Scale bar, 5 μm. **f** Flow cytometric analysis of intracellular Bcl2 expression level in WT or *Tbkbp1*-KO thymic NKT cells cultured for 2 days with medium control, IL-15, or IL-15 plus the autophagy inhibitor 3MA. **g** Summary graph of flow cytometric analysis of AnnexinV^+^ apoptotic cells in enriched thymic NKT cells that were either untreated (NT) or cultured for 1 day with the indicated agents. **h**, **i** Co-IP analysis of Tbkbp1-FIP200 (**h**) or Tbkbp1-Ulk1(**i**) interaction (upper) and direct IB assays (lower) using lysates of HEK293 cells transfected with the indicated expression vectors. **j** IB analysis of the indicated proteins in IP samples of control IgG, anti-FIP200, or anti-Ulk1 or direct lysates of NKT hybridoma cells that were stimulated as indicated. **k** Schematic of Tbkbp1 WT and mutants showing the coiled-coil domains (CCs), TBK-binding domain (TBD), proline-rich domain, and zinc fingers (ZF). **l**, **m** Co-IP analysis of Ulk1 binding with the indicated Tbkbp1 mutants (upper) and direct IB assays (lower) using HEK293 cells transfected with Myc-ULk1 along with Tbkbp1 truncation mutants (**l**) and point mutants (**m**). **n** IB analysis of the indicated phosphorylated (P-) or total proteins in whole-cell lysates of Tbkbp1-knockdown NKT hybridoma cells reconstituted with a vector control (Ctrl) or the indicated Tbkbp1 point mutants, stimulated as indicated. Data are representative of three or more independent experiments. **P < 0.01; ***P < 0.001. Two-way ANOVA (**e**), Student’s *t*-test (**g**)

Autophagy is a fundamental mechanism that removes damaged mitochondria to prevent abnormal ROS production and maintain cell survival^32^. Accumulating studies have demonstrated the requirement of autophagy in NKT cell maturation, although the underlying mechanism is obscure^7–9^. Because of the aberrant ROS production in Tbkbp1-deficient NKT cells, we examined the role of Tbkbp1 in regulating autophagy. Consistent with a recent study performed with conventional T cells^33^, we found that IL-15 could stimulate autophagy in NKT cells, as revealed by generation of the modified form of LC3, LC3II, and reduction in the level of a well-defined autophagy substrate, p62 (Fig. 6a and Supplementary Fig. 6a). Importantly, the IL-15-stimulated LC3II generation and p62 reduction were largely blocked in the Tbkbp1-deficient NKT cells, suggesting a crucial role for Tbkbp1 in regulating IL-15-stimulated autophagy (Fig. 6a). Moreover, IL-15 stimulated Ulk1 phosphorylation at serine 555 (S555), a key initial step in autophagy induction^3^, and this signaling event was attenuated in *Tbkbp1*-KO NKT cells (Fig. 6b). To assure that these phenotypes were not due to developmental effect, we knocked down Tbkbp1 in an NKT hybridoma, NKT1.2, using two different *Tbkbp1* shRNAs. As seen with the primary NKT cells, IL-15 stimulated LC3II generation and p62 loss in NKT1.2 cells, which were blocked upon *Tbkbp1* knockdown (Fig. 6c and Supplementary Fig. 6b). The IL-15-stimulated p62 loss was inhibited by a lysosomal inhibitor, bafilomycin A, consistent with its degradation by the autophagy pathway (Fig. 6c and Supplementary Fig. 6b). Moreover, In the presence of bafilomycin A, the *Tbkbp1*-knockdown cells still had a lower level of LC3II than control cells, confirming a defect of the *Tbkbp1*-knockdown cells in LC3II generation (Fig. 6c and Supplementary Fig. 6b). Consistently, the IL-15-stimulated Ulk1 S555 phosphorylation was also attenuated in the *Tbkbp1*-knockdown NKT1.2 cells (Fig. 6d). We further confirmed the Tbkbp1-dependent autophagy induction by IL-15 based on formation of LC3 puncta (Fig. 6e) and staining with a commercial mitophagy dye (Supplementary Fig. 6c, d). Autophagy could also be detected by confocal imaging based on colocalization of mitochondria with lysosome^34^. Confocal assays revealed mitochondria-lysosome colocalization in WT, but not *Tbkbp1-KO*, NKT cells (Supplementary Fig. 6e), further emphasizing the role of Tbkbp1 in regulating autophagy. IL-2 also stimulated NKT cell autophagy in a Tbkbp1-dependent manner, but IL-7 only induced weak NKT autophagy in a Tbkbp1-independent manner (Supplementary Fig. 6f, g). Together, these findings establish Tbkbp1 as a crucial mediator of autophagy stimulated by the cytokines IL-15 and IL-2.

To determine the functional significance of autophagy in NKT cell survival, we examined the effect of an autophagy inhibitor, 3MA, on IL-15-induced Bcl-2 expression and survival in NKT cells. As expected, IL-15 strongly induced the expression of Bcl-2 in WT, but not Tbkbp1-deficient, NKT cells (Fig. 6f). Importantly, the autophagy inhibitor 3MA completely blocked IL-15-stimulated Bcl-2 expression in WT NKT cells, thereby erasing the differences between the WT and the Tbkbp1-deficient NKT cells (Fig. 6f). In the presence of 3MA, IL-15 also failed to prevent apoptosis in WT NKT cells, causing a high level of apoptosis in both WT and *Tbkbp1*-KO NKT cells even in the presence of IL-15 (Fig. 6g). The IL-15-induced Bcl-2 expression in WT NKT cells was also inhibited by several other autophagy inhibitors (Supplementary Fig. 6h) known to interfere with different steps in the autophagy pathway^35–37^. Autophagy inhibition by bafilomycin A in WT NKT cells also increased mitochondrial mass and erased the difference between WT and *Tbkbp1*-KO NKT cells (Supplementary Fig. 6i). Notably, bafilomycin A only moderately increased the mitochondrial mass in Tbkbp1-deficient NKT cells, which was consistent with the attenuated autophagy in these mutant cells. Thus, Tbkbp1-mediated autophagy regulation appears to contribute to IL-15-induced mitochondria function, Bcl-2 expression, and survival of NKT cells.

### Tbkbp1 regulates the autophagy and survival of NK cells

Like NKT cells, NK cells express high levels of the IL-2/IL-15R and rely on IL-15 for survival and maturation^38,39^. We thus examined whether Tbkbp1 also played a similar role in NK cells. Compared to WT control mice, the *Tbkbp1*-KO mice had a significant reduction in the frequencies and absolute numbers of splenic NK cells, although the BM NK cells were only moderately affected (Supplementary Fig. 7a). NK cell maturation is defined into four stages based on their surface expression of CD11b and CD27, including stage 1 (CD11b^low^CD27^low^), stage 2 (CD11b^low^CD27^high^), stage 3 (CD11b^high^CD27^high^), and stage 4 (CD11b^high^CD27^low^)^40^. Tbkbp1 deficiency reduced the frequency of stage 4 NK cells and currently increased frequencies of early stages, suggesting a role for Tbkbp1 in regulating NK cell maturation or survival (Supplementary Fig. 7b). Indeed, the Tbkbp1 deficiency impaired IL-15-stimulated NK cell survival (Supplementary Fig. 7c) as well as IL-15-induced autophagy, as revealed by attenuated LC3II generation and p62 degradation (Supplementary Fig. 7d). On the other hand, Tbkbp1 deficiency had no obvious effect on IL-15-induced survival or autophagy of CD8 memory T cells (Supplementary Fig. 7e, f). This result was consistent with the much weaker expression of Tbkbp1 in conventional T cells than in NK and NKT cells (Fig. 1a, b). Collectively, these results demonstrate an important role for Tbkbp1 in regulating IL-15-stimulated autophagy and survival of NK and NKT cells.

### Tbkbp1 associates with the Ulk1-FIP200 complex

In search of the molecular mechanism by which Tbkbp1 regulates IL-15-stimulated autophagy, we found that one of the Tbkbp1-binding proteins identified by affinity capture-mass spectrometry was FIP200 (BioGrid), an essential component of the Ulk1 autophagy initiation complex^41,42^. We confirmed the specific Tbkbp1/FIP200 interaction by co-immunoprecipitation (co-IP) assays (Fig. 6h). Consistent with the FIP200-Ulk1 association, Tbkbp1 also interacted with Ulk1 in transfected cells and was co-precipitated with both FIP200 and Ulk1 under endogenous conditions (Fig. 6i, j). Tbkbp1 contains several domains, including a TBK-binding domain (TBD) (Fig. 6k) that is also present in several other TBK1-binding proteins^2^. Interestingly, the TBD, located between amino acids 280 and 330 of Tbkbp1, was required for Tbkbp1/Ulk1 interaction (Fig. 6l, m). Mutation of two conserved residues (Q315 and L319) in the Tbkbp1 TBD severely crippled the Tbkbp1-Ulk1 association, whereas mutation of another residue (Y298) did not affect the binding (Fig. 6m). Consistently, Tbkbp1 Y298A but not the Ulk1 interaction-defective Tbkbp1 mutants, Q315A and L319A, was able to rescue the defect of *Tbkbp1*-knockdown NKT hybridoma cells in autophagy induction (Fig. 6n). These findings suggest that Tbkbp1 may regulate autophagy induction by physically interacting with Ulk1 autophagy initiation complex.

### Tbkbp1 facilitates Ulk1 activation by antagonizing mTORC1

**Fig. 7:**
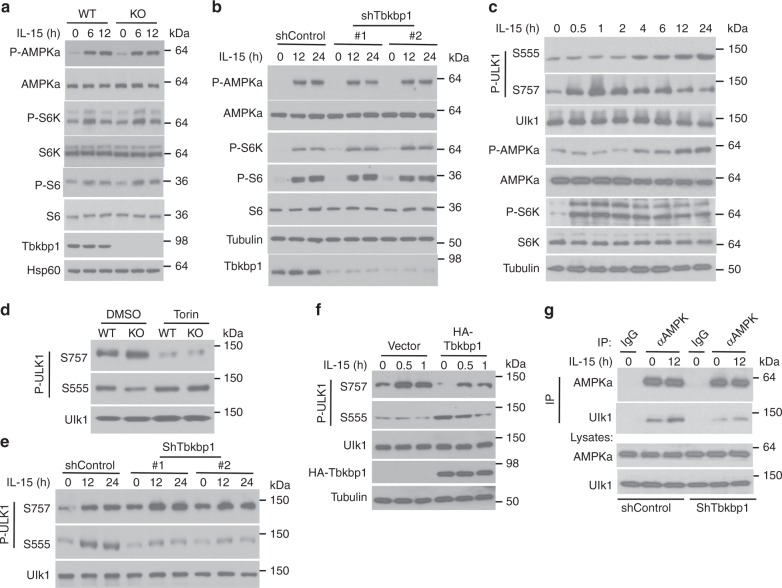
Tbkbp1 facilitates AMPKa-mediated Ulk1 activation by antagonizing mTORC1. **a**, **b** IB analysis of the indicated phosphorylated (P-) and total proteins in whole-cell lysates of IL-15-stimulated WT and *Tbkbp1*-KO thymic NKT cells (**a**) or NKT hybridoma cells transduced with a non-silencing control shRNA or two different Tbkbp1-specific shRNAs (**b**). **c** IB analysis of the indicated phosphorylated (P-) and total proteins in whole-cell lysates of IL-15-stimulated NKT hybridoma cells. **d**, **e** IB analysis of phosphorylated Ulk1 on Ser757 or Ser555 using WT or *Tbkbp1*-KO NKT cells incubated for 60 min with the mTOR inhibitor Torin or solvent control DMSO (**d**) or IL-15-stimulated NKT hybridoma cells transduced with a nonsilencing control shRNA or two different Tbkbp1-specific shRNAs (**e**). **f** IB analysis of phosphorylated Ulk1 on Ser757 or Ser 555 in IL-15-stimulated NKT hybridoma cells transduced with an empty vector or HA-Tbkbp1 expression vector. **g** Co-IP analyses of AMPKα-Ulk1 interactions (upper panels) and direct IB analyses of the indicated proteins (lower panels) in NKT hybridoma cells transduced with a control or Tbkbp1-specific shRNA (#1) incubated with IL-15 as indicated. IgG was used as a negative control for IP. Data are representative of three or more independent experiments

Ulk1 activation is a crucial step in autophagy induction, which is reciprocally regulated by two kinases: mTORC1 and AMPKa^2,3–5^. AMPKa activates Ulk1 by phosphorylating Ulk1 at S555 and additional activation sites, whereas mTORC1 inhibits Ulk1 activation by phosphorylating Ulk1 at S757 and, thereby, inhibiting Ulk1-AMPKa interaction and AMPKa-mediated Ulk1 phosphorylation. Autophagy induction by nutrient deprivation involves mTORC1 inactivation and AMPKa activation, switching Ulk1 from mTORC1-suppressed state to AMPKa-activated state^4^. Consistent with its ability to induce autophagy, IL-15 stimulated the activation of AMPKa in primary NKT cells and NKT hybridoma cells (Fig. 7a, b). Surprisingly, in contrast to nutrient deprivation, IL-15 did not inhibit but rather activated the AMPKa-opposing kinase mTORC1, as revealed by the phosphorylation of its substrate S6K and the downstream ribosomal protein S6 (Fig. 7a, b). Moreover, *Tbkbp1* knockout or knockdown had no effect on IL-15-stimulated activation of AMPKa and mTORC1 (Fig. 7a, b). A more detailed time-course analysis revealed that IL-15 stimulated rapid activation of mTORC1 but delayed activation of AMPKa, which was associated with a shift of Ulk1 phosphorylation from S757 to S555 (Fig. 7c). These findings raised the question of how the Ulk1 phosphorylation events were modulated.

Because of the physical interaction between Tbkbp1 and FIP200/Ulk1, we surmised that Tbkbp1 might act on Ulk1 to regulate its phosphorylation by AMPKα or mTORC1. Indeed, *Tbkbp1* knockout or knockdown profoundly enhanced Ulk1 S757 phosphorylation under both basal and IL-15-stimulated conditions, which was associated with reduced Ulk1 phosphorylation at S555 (Fig. 7d, e). Consistent with a prior study^4^, the Ulk1 S757 phosphorylation was mediated by mTORC1, since it was blocked by the mTOR inhibitor Torin (Fig. 7d). Importantly, Torin-mediated inhibition of S757 phosphorylation promoted Ulk1 S555 phosphorylation and overrode the suppression of Ulk1 S555 phosphorylation in Tbkbp1-deficient NKT cells (Fig. 7d). In line with these findings, overexpression of Tbkbp1 inhibited Ulk1 S757 phosphorylation and concomitantly promoted Ulk1 S555 phosphorylation (Fig. 7f). It is known that the mTORC1-mediated Ulk1 S757 phosphorylation inhibits Ulk1-AMPKα interaction^4^. Consistently, *Tbkbp1* knockdown enhanced Ulk1 S757 phosphorylation (Fig. 7e) and concomitantly inhibited Ulk1-AMPKα interaction (Fig. 7g). These results suggest that Tbkbp1 facilitates IL-15-stimulated autophagy by antagonizing mTORC1-mediated Ulk1 S757 phosphorylation and, thereby, promoting Ulk1-AMPKα association and AMPKα-mediated Ulk1 S555 phosphorylation.

### Tbkbp1 recruits an mTORC1-opposing phosphatase to Ulk1

**Fig. 8:**
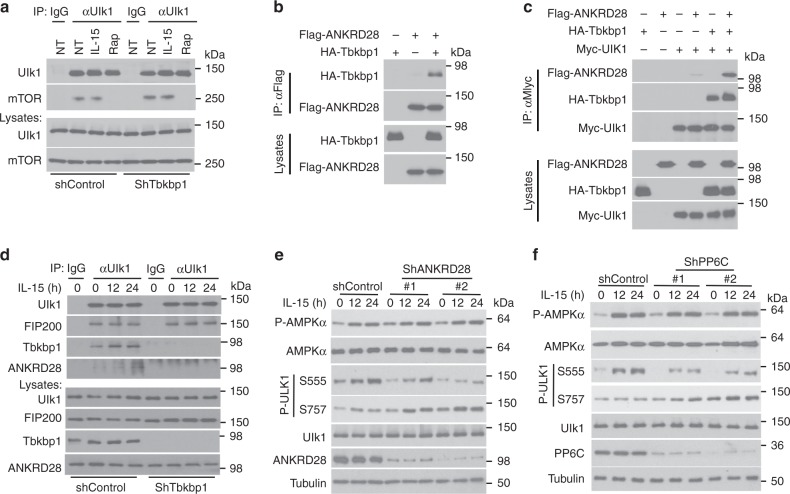
Tbkbp1 recruits PP6 to Ulk1 to oppose mTORC1-mediated Ulk1 phosphorylation. **a** Co-IP analysis of mTOR-Ulk1 interactions (upper panels) and direct IB analysis of the indicated proteins (lower panels) in NKT hybridoma cells transduced with a control or Tbkbp1-specific shRNA, either not treated (NT) or incubated with IL-15 or the mTORC1 inhibitor rapamycin (Rap). IgG was used as a negative control for IP. **b** Co-IP analysis of Tbkbp1-ANKRD28 interactions and direct IB assays using lysates of HEK293 cells transfected with the indicated expression vectors. **c** Co-IP analysis of ULK1 association with Tbkbp1 and ANKRD28 using lysates of HEK293 cells transfected with the indicated expression vectors. **d** Co-IP analysis of Ulk1 interaction with FIP200, Tbkbp1, and ANKRD28 using lysates of IL-15-stimulated NKT hybridoma cells transduced with a control or Tbkbp1-specific shRNA. IgG was used as a negative control for IP. **e**, **f** IB analysis of the indicated phosphorylated (P-) and total proteins in whole-cell lysates of IL-15-stimulated NKT hybridoma cells transduced with a nonsilencing control shRNA, two different ANKRD28-specific shRNAs (**e**) or two different PP6c-specific shRNAs (**f**). Data are representative of three independent experiments

Since Tbkbp1 deficiency enhanced mTORC1-mediated Ulk1 S757 phosphorylation without promoting mTORC1 activation (Fig. 7b–d), we examined whether Tbkbp1 regulates the association of mTORC1 with Ulk1. To our surprise, *Tbkbp1* knockdown did not significantly enhance the binding of mTORC1 to Ulk1 (Fig. 8a), suggesting the involvement of a different mechanism. In this regard, mTORC1-mediated protein phosphorylation is subject to regulation by phosphatases, especially protein phosphatase 6 (PP6)^43^. Notably, proximity label-mass spectrometry analysis identified physical association between Tbkbp1 and a regulatory subunit of PP6, ANKRD28 (BioGrid database). We confirmed the strong Tbkbp1-ANKRD28 interaction by coIP assays (Fig. 8b). Moreover, although ANKRD28 did not interact with Ulk1 directly, these two proteins formed a complex in the presence of Tbkbp1, suggesting that Tbkbp1 functioned as an adapter to recruit ANKRD28 to Ulk1 (Fig. 8c). Moreover, endogenous ANKD28 was recruited to the Ulk1-FIP200 complex, along with Tbkbp1, in IL-15-stimulated NKT hybridoma cells, and this inducible recruitment was abolished in the *Tbkbp1*-knockdown cells (Fig. 8d). These results suggested the intriguing possibility that Tbkbp1-mediated Ulk1 regulation involves recruitment of PP6. In further support of this idea, silencing ANKRD28 with 2 different shRNAs caused a profound increase in Ulk1 S757 phosphorylation and a concomitant decrease in Ulk1 S555 phosphorylation (Fig. 8e). This result was more striking for ANKRD28 knockdown with shRNA#2 than that with shRNA#1, which was consistent with the higher knockdown efficiency of shRNA#2 (Fig. 8e). Similarly, PP6C knockdown also promoted Ulk1 S757 phosphorylation and reduced Ulk1 S555 phosphorylation (Fig. 8f). Together, these results suggest that Tbkbp1-mediated regulation of Ulk1 activation may involve recruitment of PP6 to Ulk1.

## Discussion

The results presented in this paper established Tbkbp1 as a crucial regulator of NKT cell survival and development. Tbkbp1 deficiency caused a selective loss of IFNγ-producing NKT1 cells, resulting in a relative increase in the frequency of IL-4-producing NKT2 cells. The enhanced steady-state IL-4 production in turn contributed to increased generation of memory-like CD8^+^ T cells in the *Tbkbp1*-KO mice. Tbkbp1 had a cell-intrinsic role in NKT cell regulation and acted by mediating the survival signal of IL-15. Our data revealed that IL-15 induces NKT cell autophagy via a Tbkbp1-dependent mechanism.

Autophagy activation has been extensively studied as a response to stress conditions, such as nutrient deprivation, but how autophagy is induced during physiological processes is still less well understood. Our present study provided an example of physiological autophagy induction in the immune system. Unlike nutrient deprivation, which triggers autophagy by inactivating mTORC1^4^, the IL-15-mediated autophagy induction occurred along with mTORC1 activation. Tbkbp1 antagonized the autophagy-inhibitory function of mTORC1 by preventing mTORC1-mediated Ulk1 phosphorylation at an inhibitory residue (S757), thereby facilitating Ulk1 activation by AMPKa. This function of Tbkbp1 was specific and involved physical interaction with the Ulk1-FIP200 complex and recruitment of an mTORC1-opposing phosphatase, PP6. Thus, Tbkbp1 enables autophagy induction by IL-15 without inactivation of mTORC1.

We showed that Tbkbp1 deficiency impaired IL-15-induced expression of Bcl2 and Bcl-XL and survival of NKT cells without compromising the major signaling events, including activation of STAT5 and AKT. We obtained evidence that autophagy induction is an integral part of the survival function of IL-15, since pharmacological inhibition of autophagy impaired IL-15-induced Bcl2 expression and apoptosis inhibition. Of note, mice deficient in autophagy have selective loss of stage 3 NKT (or NKT1) cells^8^, a phenotype that is similar to that of the *Tbkbp1*- or *Il15*-deficient mice. Autophagy also plays a role in the regulation of NK cells and conventional T cells^6–10^. We found that Tbkbp1 was required for IL-15-mediated autophagy and survival of NK cells but not of memory CD8^+^ T cells. One possible reason for such functional selectivity of Tbkbp1 is its abundant expression in NK and NKT cells and relatively weak expression in conventional T cells.

Tbkbp1 deficiency had little or no effect on the development of NK1.1^–^ (stages 1 and 2) NKT cells but caused a severe loss of the NK1.1^+^CD44^+^ (stage 3) NKT cells. This phenotype is reminiscent of the mice with deficiencies in IL-15 signaling, which have selective loss of stage 3 NKT cells^18,20^. While these results may suggest a role for Tbkbp1 in regulating NKT terminal maturation, recent studies suggest that the previously defined stage 2 NKT cells actually contain the mature NKT2 and NKT17 sublineages, whereas the stage 3 cells form the mature NKT1 cells. Our data, based on analysis of the lineage marker IL-17Rb and transcription factors, suggest an essential role for Tbkbp1 in regulating the homeostasis and survival of NKT1, but not NKT2 or NKT17, cells. These results are consistent with the previous finding that NKT1 cells, but not NKT2 or NKT17 cells, abundantly express IL-15Rb and rely on IL-15 for survival and homeostasis^19,20^. A recent study further suggests that the survival of NKT17 cells is mediated by IL-7 but not IL-15^21^. Consistently, we found Tbkbp1 to be required for NKT cell survival induced by IL-15 but not by IL-7.

The *Tbkbp1*-KO mice generally resemble *Il15*-KO mice in the phenotype of NKT cell development, but there seem to be some minor differences between these two mutant strains. Although both *Tbkbp1*-KO and *Il15*-KO mice have a drastically reduced absolute number of stage 3 NKT cells, the former also have a more severe reduction in the percentage of this NKT population. While this could be due to different experimental conditions, it is also likely that Tbkbp1 may play a crucial, but not the only, role in mediating IL-15-induced NKT cell survival. One possibility is that Tbkbp1 may also mediate the signaling function of additional homeostatic cytokines, such as other members of the γc cytokine family. In support of this possibility, we found that Tbkbp1 was also required for IL-2-induced NKT cell autophagy and survival. Future studies will examine the role of Tbkbp1 in regulating the signaling function of additional cytokines in NKT cells.

## Methods

### Mice

*Tbkbp1*-targeted mice, *Tbkbp1*^tm1a(EUCOMM)Wtsi^ (in C57BL/6 N background), were generated at Knockout Mouse Project (KOMP) by targeting exon 4 of *Tbkbp1* gene using a FRT-LoxP vector. Germline *Tbkbp1*-KO mice were generated by crossing the *Tbkbp1*-targeted mice with EIIA-Cre mice (The Jackson Laboratory). Heterozygous (Tbkbp1^+/–^) mice were bred to generate age matched WT and homozygous *Tbkbp1-KO* mice for experiments. *Tbkbp1*-flox mice were generated by crossing the *Tbkbp1*-targeted mice with FLP deleter mice (Rosa26-FLPe; 129S4/Sv background; The Jackson Laboratory), and the *Tbkbp1*-flox mice were further crossed with *Lck*-Cre mice (The Jackson Laboratory) to generate *Tbkbp1* T cell-conditional KO (*Tbkbp1-TKO*; Tbkbp1^fl/fl^Lck-Cre) and WT (Tbkbp1^+/+^Lck-Cre) mice.

C57BL/6 mice were from The Jackson Laboratory (002518). *Tbkbp1*-*Il4-*KO mice were generated by crossing the *Tbkbp1*-KO mice with *Il-4*-KO mice. Heterozygous (*Tbkbp1*^+/–^*Il4*^+/–^) mice were bred to generate age matched WT, *Tbkbp1*-KO, *Il4*-KO and *Tbkbp1*/*Il4*-double KO mice for experiments. Mice were maintained in a specific pathogen–free facility, and all animal experiments were conducted in accordance with protocols approved by the Institutional Animal Care and Use Committee of the University of Texas MD Anderson Cancer Center.

### Plasmids

pCLXSN(GFP)-HA-Tbkbp1 was generated by inserting the mouse Tbkbp1 cDNA, along with an N-terminal HA tag, into the pCLXSN(GFP) retroviral vector^44^, and the same approach was used to construct pCLXSN(GFP)-based expression vectors encoding truncated forms of Tbkbp1, 1-280 and 1-330. The pCLXSN(GFP)-HA-Tbkbp1 was used as template to generate Tbkbp1 point mutants, Y298A, Q315A, and L319A, by site-directed mutagenesis using a QuickChange II Site-Directed Mutagenesis Kit (Agilent). pcDNA-myc-Ulk1 and p3xFLAG-FIP200 were purchased from Addgene. pGIPZ lentiviral vectors encoding shRNAs silencing Tbkbp1, Ankrd28 and ppp6c or a nonsilencing control shRNA were purchased from Thermo Fisher Scientific. Flag-tagged Ankrd28 was provided by Michiyuki Matsuda.

### Antibodies and reagents

Antibodies for phospho-Akt (9271, 1:1000), Phospho-Stat5 (9359, 1:1000), Stat5 (9363, 1:1000), α-Tubulin (2144,1:2000), Phospho-Ulk1 Ser555 (5869, 1:500), Phospho-Ulk1 Ser467 (4634, 1:1000), Phospho-Ulk1 Ser757 (14202, 1:1000), Ulk1 (8054, 1:1000), Phospho-AMPKα (2535, 1:1000), AMPKα (5831, 1:1000), Phospho-p70 S6K (9206, 1:1000), p70 S6K (9202, 1:1000), FIP200 (12436, 1:1000), mTOR (2972, 1:1000), and Tbkbp1 (8605, 1:1000) were from Cell Signaling Technology. Antibody for Akt1(sc-5298,1:1000), c-Myc(sc-40, 1:1000), Ankrd28(sc-393032,1:1000) and  Hsp60(sc-13115, 1:2000) was from Santa Cruz Biotechnology. Antibody for PPP6C (A300-844A, 1:1000) was obtained from Bethyl Laboratories, Inc. Horseradish peroxidase–conjugated anti-HA antibody (3F10, 1:2000) was purchased from Roche. Anti-LC3 antibody (L7543, 1:1000) and horseradish peroxidase–conjugated anti-Flag antibody (M2, 1:5000) were purchased from Sigma-Aldrich. Horseradish peroxidase–conjugated anti-c-Myc (MCA2200P, 1:1000) was from Bio-Rad. Horseradish Peroxidase-conjugated Donkey anti-mouse IgG (715-035-151, 1:10,000), Horseradish Peroxidase-conjugated Goat anti-mouse IgG light chain (115-035-174, 1:2000), Horseradish Peroxidase-conjugated Donkey anti-rabbit IgG (711-035-152, 1:10,000), and Horseradish Peroxidase-conjugated Mouse anti-rabbit IgG light chain (211-032-171, 1:2000) were purchased from Jackson ImmunoResearch.

Fluorescence-labeled antibodies for murine (m) CD4 (L3T4, 1:300), mCD8 (53-6.7, 1:300), CD44 (IM7, 1:300), mTCRβ (H57-597, 1:300), mCD45.1 (A20, 1:300), mCD45.2 (104, 1:300), mNK1.1 (PK136, 1:300), PLZF (Mags.21F7, 1:200), T-bet (4B10, 1:200), mIL-17RB (MUNC33, 1:300), mCD122 (TM-beta1, 1:3200), mCXCR3(CXCR3-173, 1:300), mEomes (Dan11mag, 1:200), mBcl-2 (10C4, 1:200), mCD1d (WTH-2, 1:300), mCD27 (LG.7F9, 1:300), mCD11b (M1/70, 1:300), hCD4 (S3.5, 1:300), hCD8 (3B5, 1:300), IL-4 (11B11, 1:300), and IFN-γ (XMG1.2, 1:300) were purchased from eBioscience. Fluorescence-labeled antibodies for murine mBcl-XL (7B2.5, 1:200) was from SouthernBiotech. Fluorescence-labeled antibodies for murine mRORγt (Q31-378, 1:200) was from BD. Fluorescence-labeled antibodies for human TCR α/β Antibody (IP26, 1:300), human TCR Vα24-Jα18 (6B11, 1:300) were purchased from Biolegend.

α-Galactosyl Ceramide (α-GalCer; KRN7000) was from Avanti Polar Lipids (867000 P). Mouse CD1d-tetramer loaded with the α-GalCer analog PBS57 (mCD1d PBS57) and unloaded mouse CD1d-tetramer control were provided by the NIH Tetramer Core Facility. FITC Annexin-V Apoptosis Detection Kit (556547) was from BD Bioscience, and Mitophagy Detection Kit (MD01-10) was from Dojindo Molecular Technologies. Nicotinamide (NAM; N0636), 3-Methyladenine (3MA; M9281), Bafilomycin A (B1793), Thapsigargin (T9033), and DL-a-difluoromethylornithine (DFMO; D193) were purchased from Sigma-Aldrich. Mouse IL-2 (402-ML), mouse IL-7 (407-ML), mouse IL-15 (447-ML), and human IL-2 (202-IL) were purchased from R&D. MitoTracker™ Green FM (M7514), MitoSOX™ Red Mitochondrial Superoxide Indicator (M36008), MitoTracker™ Red CMXRos(M7512), CellROX™ Deep Red Flow Cytometry Assay Kit (C10491) and LysoTracker™ Deep Red (L12492) were from Thermo Fisher Scientific. Monensin was from eBioscience (00–4505-51). PMA (P1585) and Ionomycin (I0634) were from Sigma-Aldrich.

A list of primers is included in Supplementary Table 1.

### Flow cytometry and intracellular cytokine staining

Suspensions of thymocytes, splenocytes and liver cells were prepared as described^44,45^. The cells were stained with the indicated fluorescence-conjugated antibodies and subjected to flow cytometry analysis as described^44^ using LSR II (BD). NKT cells were detected using pacific blue-labeled TCRβ antibody and APC- or PE-labeled CD1d-tetramer loaded with PBS57. The unloaded CD1d-tetramer was used as negative control. For intracellular cytokine staining (ICS), NKT and T cells were stimulated for 4 h with PMA plus ionomycin in the presence of a protein transport inhibitor, monensin (1:1,000), and then subjected to ICS and flow cytometry analyses. Gating strategies are shown in Supplementary Fig. 8 and 9. The data were analyzed using FlowJo software. Mitochondrial mass, mitochondria-associated ROS, and mitochondrial membrane potential were measured by flow cytometry following staining of cells at 37 °C with MitoTracker green (50 nM, 30 min), MitoSOX (2.5 μM, 30 min) and MitoTracker Red CMXRos (100 nM, 45 min), respectively. ROS was also measured by flow cytometry following staining of cells with CellROX™ Deep Red Kit according to the manufacturer’s instructions (Thermo Fisher Scientific).

### Autophagy detection by confocal microscopy

Cells were either not treated or stimulated with IL-15 for 24 h and fixed with 4% (w/vol) paraformaldehyde in PBS for 10 min at room temperature. The fixed cells were washed three times with PBS and then permeabilized with digitonin (50 μg/ml in PBS) for 5 min at room temperature and blocked with 10% goat serum in PBS. For autophagy detection, the cells were incubated with anti-LC3 antibody (in 10% goat serum) overnight at 4 °C followed by incubation with goat anti-rabbit IgG (H+L) secondary antibody conjugated with Alexa fluor 555 (Invitrogen) for 60 min. Slides were mounted in antifade reagent with DAPI (Invitrogen, P36931), and pictures were taken with an SP5 RS confocal microscope (Leica) and analyzed by SlideBook 5.0 software.

For mitochondria and lysosome colocalization analysis, sorted NKT cells from WT or *Tbkbp1*-KO mice were incubated with 100 nM MitoTracker™ Green and 50 nM LysoTracke Deep Red for 30 min at 37 °C. Cells were washed three times with PBS. Slides were mounted in antifade reagent with DAPI (Invitrogen, P36931), and pictures were taken with an SP5 RS confocal microscope (Leica) and analyzed by SlideBook 5.0 software.

### NKT and NK cell isolation and stimulation

Thymocytes were prepared from young adult mice (6–8 week old) and incubated with PE-labeled anti-CD1d-tettamer antibody for isolation of NKT cells using anti-PE magnetic beads. The NKT cells were further purified by flow cytometric cell sorting based on TCRβ^+^ CD-1d tetramer^+^ staining. The cells were stimulated with IL-15 and subjected to IB analysis of cell signaling and autophagy. Where indicated, NKT cells were also enriched from thymocytes using anti-CD8 magnetic beads by depleting CD8^+^ thymocytes, and the enriched population was cultured with IL-15 followed by flow cytometric analysis of Bcl-2 and Bcl-xl expression, mitochondria content, mitochondrial potential, ROS concentration, proliferation, and apoptosis.

For NK cell isolation, splenocytes were prepared from young adult mice (6–8 week old), incubated with biotinylated monoclonal antibodies for CD4, CD8, CD19, MHC Class II, and Ly-6G and anti-biotin microbeads (Miltenyl) to deplete unwanted cells by negative selection. The NK cells were further purified by flow cytometric cell sorting based on TCRβ^–^NK1.1^+^ staining. Purified NK cells were stimulated with IL-15 and subjected to IB analysis of autophagy induction based on LC3 modification and Ulk1 phosphorylaiton.

### Cell proliferation and apoptosis assays

Enriched NKT Cells were stained for 5 min with carboxyfluorescein succinimidyl ester (CFSE) in PBS with a final concentration of 5 μM and then washed with ice cold RPMI-1640 medium supplemented with 10% FCS and incubated on ice for 5 min. Cells were washed twice in culture media and cultured as indicated and then subjected to flow cytometry analysis of cell proliferation based on CFSE dilution. For apoptosis assays, the cells were incubated for 15 min with FITC-annexin V and propidium iodide (PI) and subjected to flow cytometry to quantify the apoptotic cell population.

### Memory CD8^+^ T cell generation in vitro

Memory CD8^+^ T cells were generated as described^46^. In brief, naïve CD8^+^ T cells were activated using plate bound α-CD3 (5 μg/ml), soluble α-CD28 (0.5 μg/ml), and mIL-2 (100 U/mL) for 3 days. T cells were then cultured in mIL-15-supplemented medium (100 U/ml) for 3 more days with daily changes of fresh mIL-15 medium. Memory CD8 T cells were washed and starved for overnight and then were restimulated with mIL-15 for apoptosis and IB assays.

### Human T cell and NKT cell culture

Human peripheral blood mononuclear cells (PBMCs) were cultured in RPMI 1640 medium (Gibco) supplemented with 10% human serum (Gemini Bio, 100-512), 10 mM HEPES buffer solution, 2 mM GlutaMAX, 1 mM sodium pyruvate, 5.5 mM 2-ME, 100 unit/ml penicillin, and 100 g/ml streptomycin. For preparing conventional T cells, PBMCs were cultured with 1000 U hIL-2 at a concentration of 2 × 10^6^ cells/well in a 24-well plate by changing media every 2 days. On day 7, CD4^+^ and CD8^+^ T cells were analyzed by flow cytometry and sorted for RNA isolation and IB assays. For NKT cell culture, PBMCs were cultured with 100 U hIL-2 and 100 ng/ml α-Galcer at a density of 2 × 10^6^ cells/well in a 24-well culture plate by changing media every 2 days. On day 14, NKT cells were analyzed by flow cytometry and sorted for RNA isolation and immunolbot assays.

### Cell culture and viral transduction

Vα14i NKT cell hybridoma 1.2 (NKT1.2) was provided by M Kronenberg (La Jolla Institute for Allergy and Immunology, La Jolla, California) and described previously^47^. For gene silencing, lentiviral particles were prepared by transfecting HEK293 cells (ATCC) with pGIPZ lentiviral vectors encoding specific shRNAs or control shRNAs along with packaging plasmids. The packaged viruses were then used to infect NKT1.2 cells, followed by selection of the infected cells by flow cytometric cell sorting based on GFP expression (pGIPZ vector carries the GFP gene). The infected cells were stimulated with IL-15 for signaling and autophagy analyses. For overexpression studies, the cells were infected with retroviral vectors for the indicated cDNAs.

### IB and coIP assays

Whole-cell lysates were prepared and subjected to IB and coIP assays as described previously^48^. The density of the protein bands in photographic films was quantified by densitometry using the ImageJ software, and the level of LC3II and p62 proteins was presented as ratios to that of loading control (tubulin). SD is calculated from the mean of three independent experiments. Statistical analysis was performed by two-way ANOVA with Bonferroni multiple comparison test. Uncropped gel images are shown in Supplementary Fig. 10–14.

### BM adoptive transfer

BM cells isolated from *Tbkbp1-KO* or WT mice (CD45.2^+^) were mixed with BM cells from B6.SJL (CD45.1^+^) mice (in 1:4 ratio) and adoptively transferred into irradiated (950 rad) *Rag1*-KO mice. After 6 week, the chimeric mice were sacrificed for analysis of NKT cell development and memory-like CD8^+^ T cells.

### Tumor models

Murine B16 melanoma cells expressing the surrogate tumor antigen chicken ovalbumin (B16-OVA) were cultured in DMEM supplemented with 10% FBS, and the tumor cells (5 × 10^5^) were injected s.c. into 8-week-old WT or *Tbkbp1-KO* mice. The challenged mice were monitored for tumor growth, and tumor size was expressed as tumor area. For survival rate calculation, mice with a tumor size reaching 225 mm^2^ were considered lethal and sacrificed based on the protocol approved by the Institutional Animal Care and Use Committee of the University of Texas MD Anderson. To minimize individual variations, age- and sex-matched (mostly littermate) WT and *Tbkbp1-KO* mice were used.

For depletion of CD8^+^ T cells, 8-week-old WT or Tbkbp1-KO mice challenged with murine B16 melanoma cells were administered i.p. with rat anti-mouse CD8α (clone 53–6.7) antibody or isotype control rat IgG2a (2A3) at the dose of 150 μg per mouse on days −2, 0, 2, 4, 6, 8, and 10. The challenged mice were monitored for tumor growth and sacrificed on day 12 to check the efficiency of CD8 T-cell depletion by flow cytometric analysis of draining lymph node cells.

### Statistical analysis

Statistical analysis was performed using Prism software (GraphPad Software 6.0). The Kolmogorov–Smirnov test was used to tests for normal distribution of the data. If the samples were normally distributed, unpaired two-tailed Student’s *t*-test was used to determine the statistical difference between two groups. For comparison of more than two groups, one-way ANOVA followed by Bonferroni multiple comparisons post-test were performed. If the samples were not normally distributed, the Mann-Whitney test was performed for two groups, Two-way ANOVA with Bonferroni multiple comparison test was used for B16-OVA tumor growth. Kaplan-Meier analyses was used and the log-rank Mantel-Cox test was employed to determine any statistical difference between the survival curves of two groups. All data are presented as mean ± SD. A *p* value <0.05 was considered significant, and the level of significance expressed as follows: *P < 0.05; **P < 0.01; ***P < 0.001. The number of animals used (*n*), and the specific statistical tests used are indicated for each experiment in the figure legends.

### Data availability

The datasets generated during the current study are available from the corresponding author on reasonable request.

## Electronic supplementary material

Supplementary Information
