# Supplementary material for: TBK-binding protein 1 regulates IL-15-induced autophagy and NKT cell survival
Source: Nat Commun. 2018 Jul 18;9:2812. doi: 10.1038/s41467-018-05097-5 (PMC6052109; doi:10.1038/s41467-018-05097-5)
Supplement: Supplementary file 1 — Supplementary Information [file 41467_2018_5097_MOESM1_ESM.pdf]

TBK-binding protein 1 regulates IL-15-induced autophagy and NKT cell survival

Zhu et al

Supplementary Figures 1-14
Supplementary Table 1

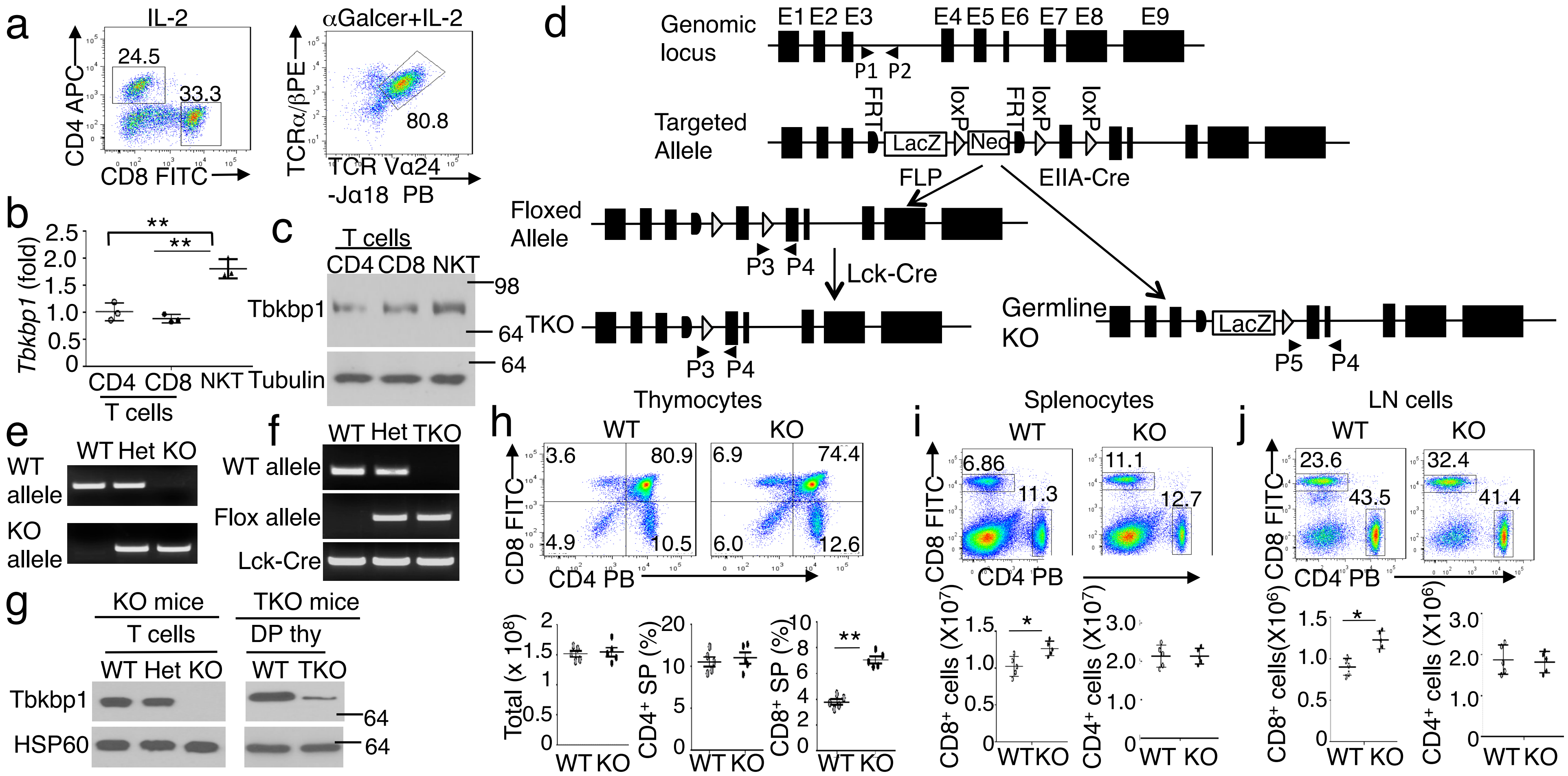

Supplementary Fig. 1. *Tbkbp1* deletion has no effect on thymocyte development. a, Flow cytometric analysis of T cells (left) and NKT cells (right) in human peripheral blood mononuclear cells (PBMCs) cultured *in vitro* for 7 days in the presence of IL-2 or for 14 days in the presence α-Galcer plus IL-2. **b,c**, qRT-PCR (**b**) and IB (**c**) analysis of Tbkbp1 mRNA and protein in $CD4^+$ and $CD8^+$ T cells or NKT cells sorted from the PBMCs described in **a**. **d**, Schematic picture of *Tbkbp1* gene targeting. Mice carrying the *Tbkbp1* targeted allele ($Tbkbp1^{tm1a(EUCOMM)Wtsi}$) were crossed with EIIA-Cre to generate germline *Tbkbp1*-knockout (KO) mice and with FLP deleter (Rosa26-FLPe) mice to generate *Tbkbp1*-floxed mice, which were further crossed with *Lck*-Cre to generate T cell-conditional KO (TKO) mice. **e**, Genotyping PCR analysis of germline *Tbkbp1* wildtype (WT), heterozygous (Het), and KO mice using P1/P2 and P4/P5 primer pairs for WT and KO alleles, respectively. **f**, Genotyping PCR analysis of *Tbkbp1* WT ($Tbkbp1^{+/+}$Lck-Cre), TKO Het ($Tbkbp1^{+/fl}$Lck-Cre), and TKO ($Tbkbp1^{fl/fl}$Lck-Cre) mice using P1/P2 and P3/P4 primer pairs for WT and Flox alleles, respectively. **g**, IB analysis of Tbkbp1 and control HSP60 proteins in spleen T cells of *Tbkbp1*-KO and control (WT and heterozygous) mice and $CD4^+CD8^+$ double-positive (DP) thymocytes of *Tbkbp1*-TKO and WT control mice. **h**, Flow cytometric analysis of thymocyte subpopulations showing a representative FACS plot (upper) and summary graph (lower) of total thymocyte numbers and frequency of $CD4^+$ and $CD8^+$ single positive (SP) thymocytes. **i,j**, Flow cytometric analysis of peripheral T cells in the spleen (**i**) and inguinal lymph nodes (iLN) (**j**), showing representative FACS plots (upper) and summary graphs (lower). *P<0.05; **P<0.01. One-way ANOVA (**b**), Student's t-test (**h,i,j**).

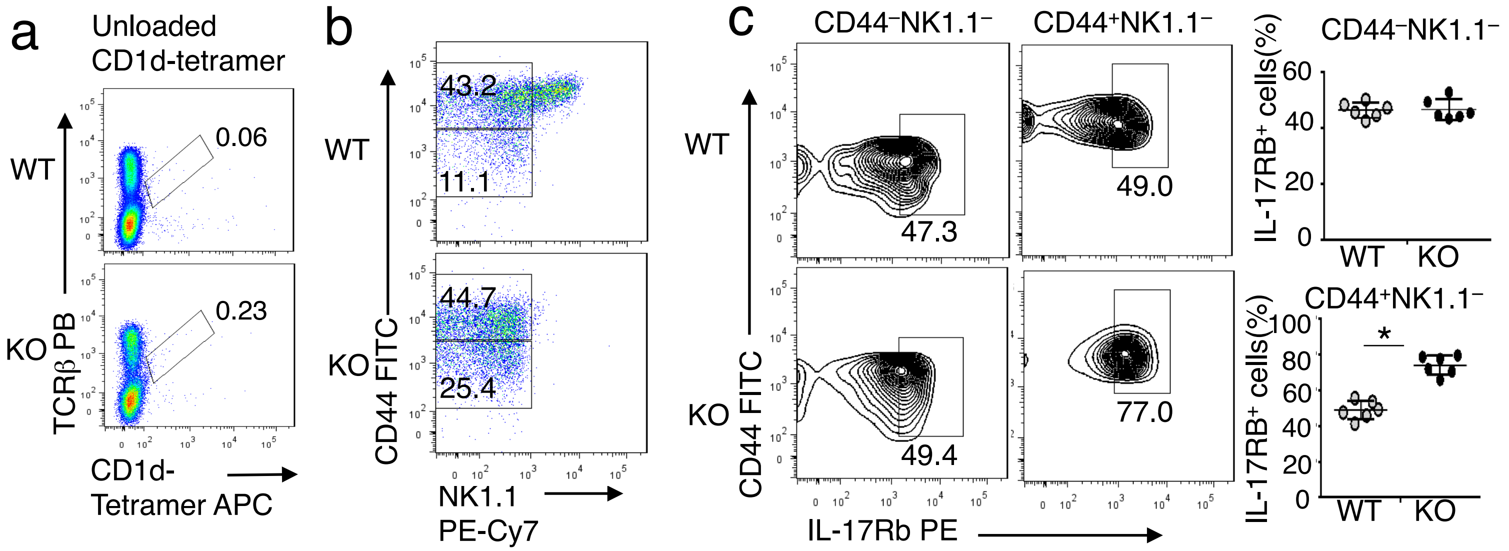

Supplementary Fig. 2. Tbkbp1 deficiency increases the frequency of IL-17Rb^{+} NKT cells in the CD44^{+}NK1.1^{-} subpopulation. a, Negative control of flow cytometric analysis of liver NKT cells using an unloaded CD1d-tetramer. **b,c**, Flow cytometric analysis of NKT cells based on CD44 and NK1.1 markers (**b**) and expression of IL-17Rb on gated thymic CD44^{-}NK1.1^{-} and CD44^{+}NK1.1^{-} NKT subpopulations, presented as representative plots and summary graphs based on multiple mice (each circle represents a mouse) (**c**). *P<0.05. Student's t-test (**c**).

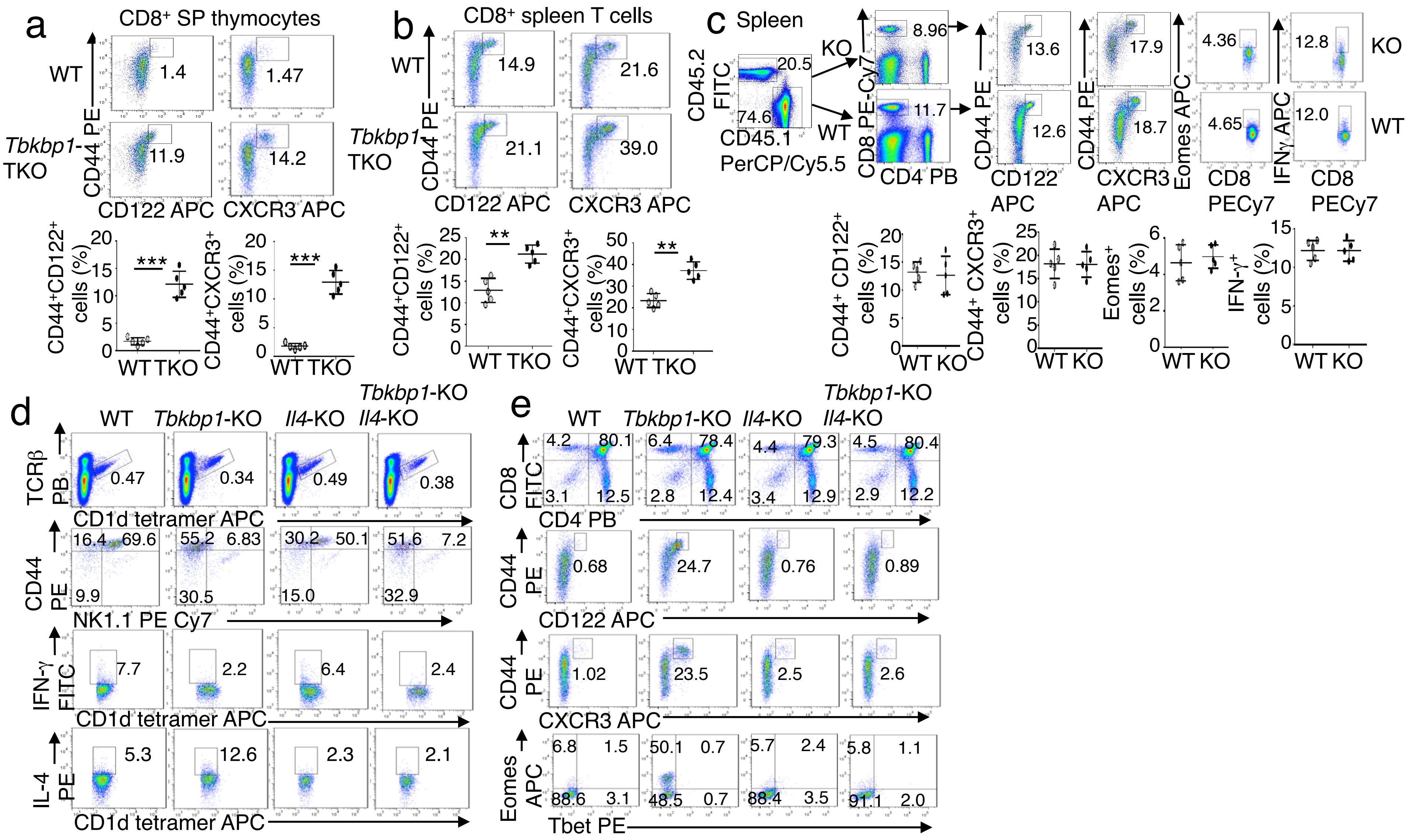

Supplementary Fig. 3. Cell-extrinsic and IL-4-dependent role of Tbkbp1 in regulating memory-like CD8^{+} T cell generation. a,b, Flow cytometric analysis of memory-like CD8^{+} T cells based on CD44^{hi}CD122^{+} and CD44^{hi}CXCR3^{+} surface markers in thymocytes (**a**) and splenocytes (**b**), presented as representative plots (upper) and summary graphs (lower). **c**,Flow cytometric analysis of memory-like CD8^{+} T cells based on expression of CD44, CD122, CXCR3, Eomes and IFN-γ in spleen CD8^{+} T cells of Rag1-KO recipient mice adoptively transferred (for 6 wk) with a mixture of BM cells derived from WT B6.SJL mice (CD45.1^{+}) and *Tbkbp1*-KO mice (CD45.2^{+}), gating on CD45.1^{+} (WT) or CD45.2^{+} (KO) cells, presented as a representative FACS plot (upper) and summary graphs based on 5 chimeric mice (lower). **d,e**, Flow cytometric analysis of NKT cell maturation and cytokine production (**d**) and memory-like CD8^{+} T cells (**e**) in the thymocytes of the indicated mouse strains. Data are representative of three independent experiments and are presented as means ± s.d. values. **P<0.01; ***P<0.001. Student's t-test (**a,b,c**).

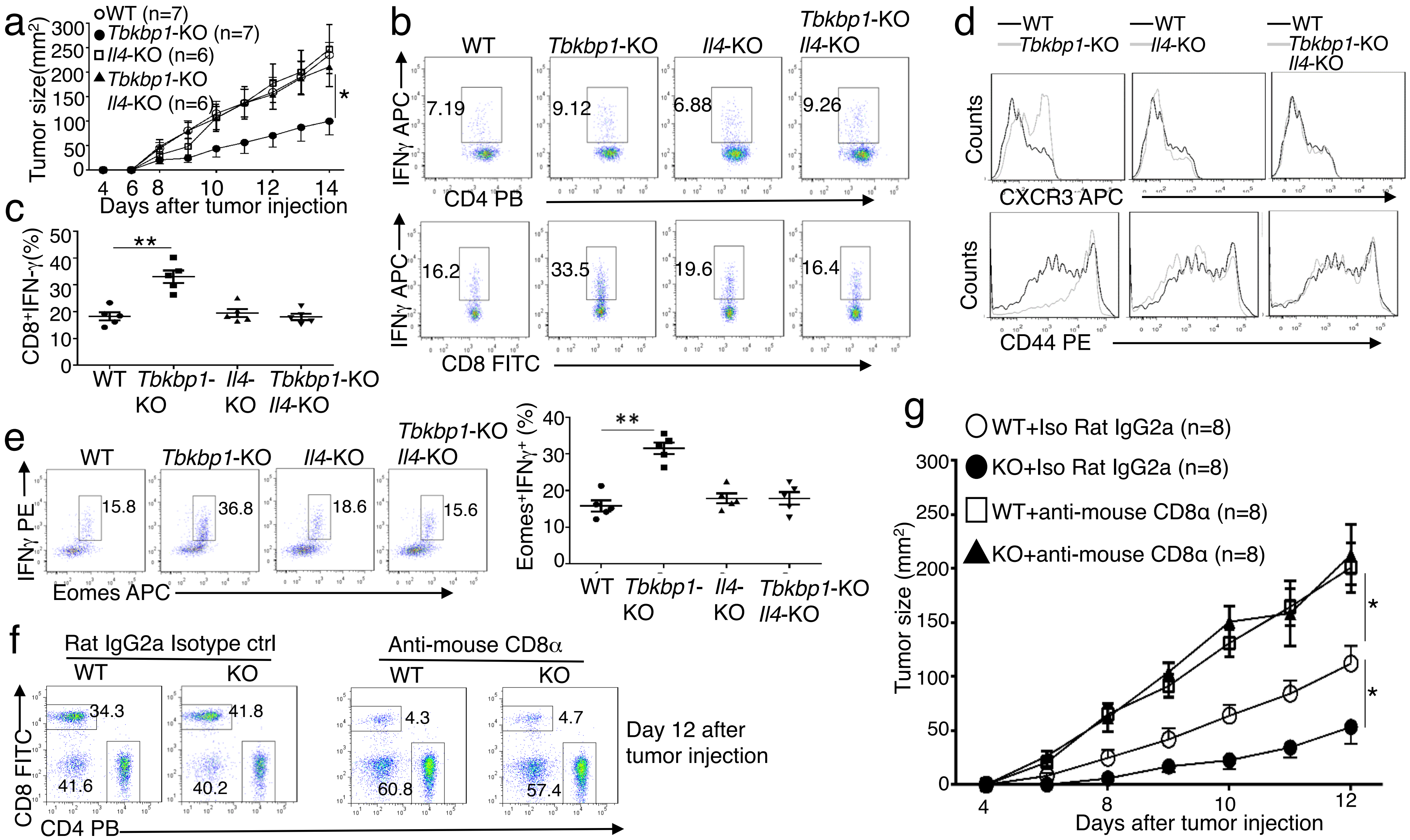

Supplementary Fig. 4. Tbkbp1 regulates CD8 T cell-dependent antitumor immunity. a-c, Tumor growth curve (**a**) and flow cytometric analysis of IFNγ-producing $CD4^+$ and $CD8^+$ effector T cells in day 14 tumors (**b**) of the indicated mice injected with B16-OVA melanoma cells, presented as representative graphs (**b**) and a summary graph (**c**). **d**, Flow cytometric analysis of expression of CXCR3 or CD44 on gated CD8 T cells in day14 tumors of the indicated mice injected with B16-OVA. **e**, Flow cytometric analysis of $IFN\gamma^+Eomes^+$ cells in tumor-infiltrating $CD8^+$ T cells of the indicated mice injected with B16-OVA (day 14), presented as representative plots (left) and a summary graph (right). **f,g**, Flow cytometric analysis of draining lymph node cells for monitoring $CD8^+$ T-cell depletion **(f)** and tumor growth curve (**g**) of WT and *Tbkbp1*-KO mice injected with B16-OVA along with a CD8α neutralizing antibody or an IgG2a isotype control. *P<0.05; **P<0.01. Two-way ANOVA (**a,g**), One-way ANOVA (**c,e**)

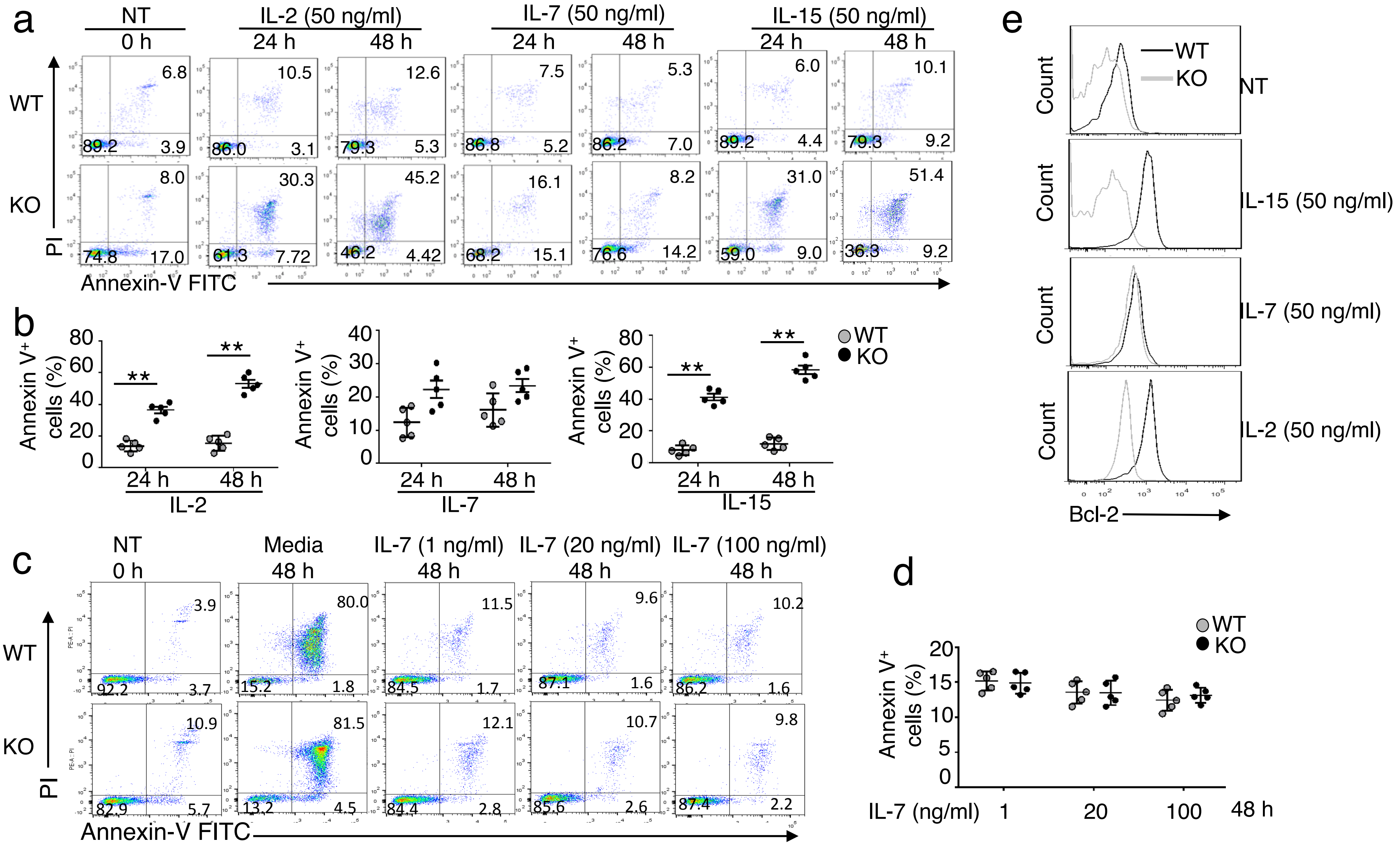

Supplementary Fig. 5. Tbkbp1 is required for NKT cell survival mediated by IL-15 and IL-2 but not by IL-7. a-d, Flow cytometric apoptosis analysis of WT or *Tbkbp1*-KO (KO) NKT cells that were either not treated (NT) or incubated with the indicated cytokines for the indicated times. Data are presented as a representative plot (**a, c**) and summary graphs based on multiple mice (each circle represents a mouse) (**b,d**). **e**, Flow cytometric analysis of intracellular Bcl2 expression level in WT or *Tbkbp1*-KO (KO) NKT cells incubated with the indicated cytokines for 48 h. Data are representative of three independent experiments and are presented as means ± SD. **, $P < 0.01$. Student's t-test (**b,d**).

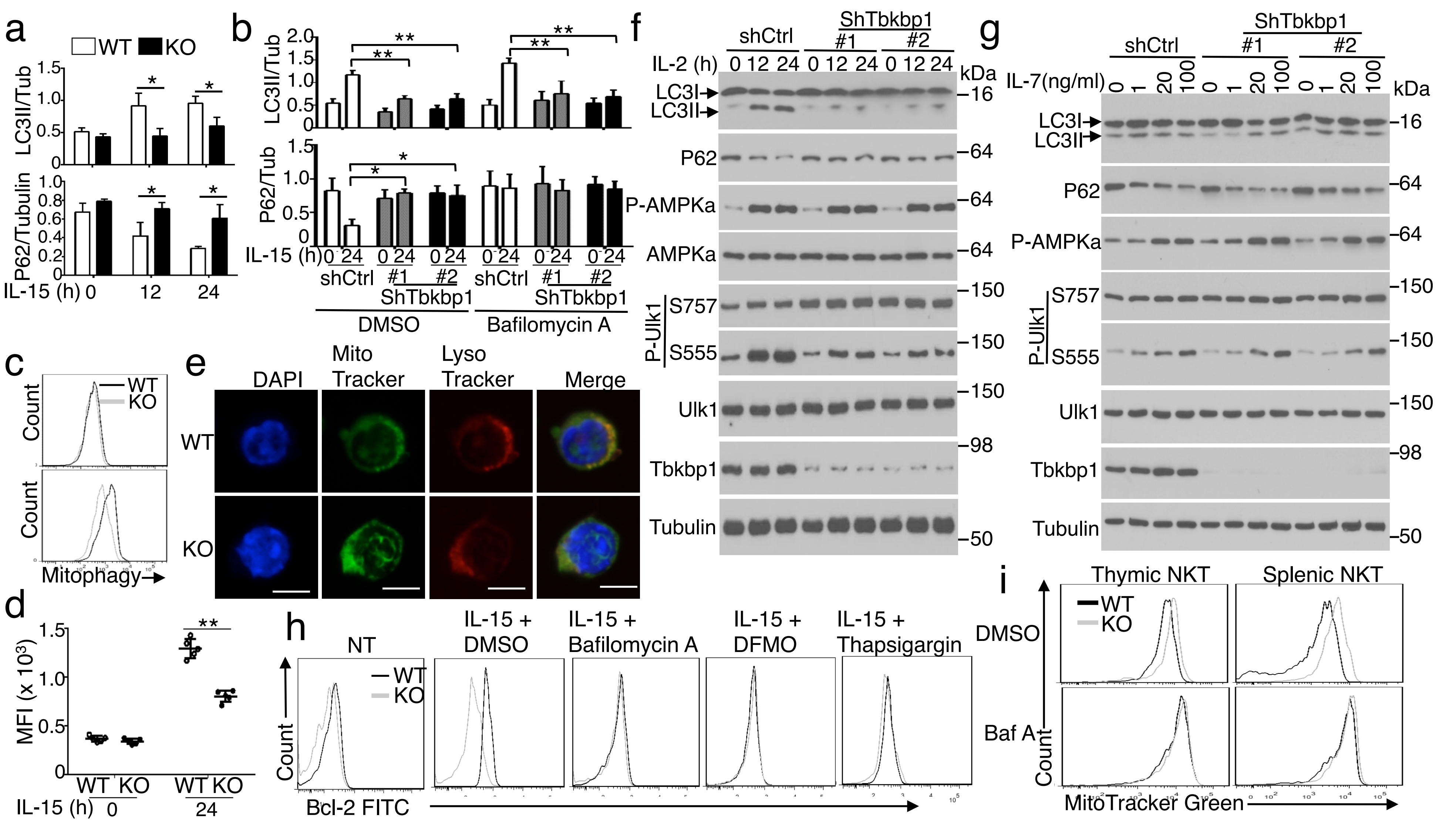

Supplementary Fig. 6. TBKBP1 is required for NKT cell autophagy induced by IL-2 and IL-15 but not by IL-7. a,b, Densitometry quantification of Fig. 6a (**a**) and Fig. 6c (**b**). **c,d,** Mitophagy analysis by flow cytometry, presented as a representative plot (**c**) and summary graph (**d**). **e,** Confocal imaging analysis of freshly isolated WT or *Tbkbp1*-KO (KO) NKT cells, showing mitochondria-lysosome colocalization in WT but not KO NKT cells. Scale bar, 5µm. **f,g,** IB analyses of the indicated phosphorylated (P-) and total proteins in whole-cell lysates of NKT hybridoma cells (NKT1.2) stably transduced with a control shRNA or two different *Tbkbp1* shRNAs following stimulation with IL-2 (50 ng/ml) for the indicated times (**f**) or with the indicated doses of IL-7 for 24 h (**g**). **h**, Flow cytometric analysis of Bcl-2 expression in WT and *Tbkbp1*-KO (KO) enriched thymic NKT cells that were either not treated (NT) or incubated for 48 h with IL-15 in the presence of DMSO or the indicated autophagy inhibitors. **i**, Flow cytometric analysis of mitochondrial mass in WT or *Tbkbp1*-KO (KO) thymic or splenic NKT cells incubated for 12 h with IL-15 in the presence of DMSO or bafilomycin A (Baf A). *P<0.05; **P<0.01. Two-way ANOVA (**a,b**), Student's t-test (**d**).

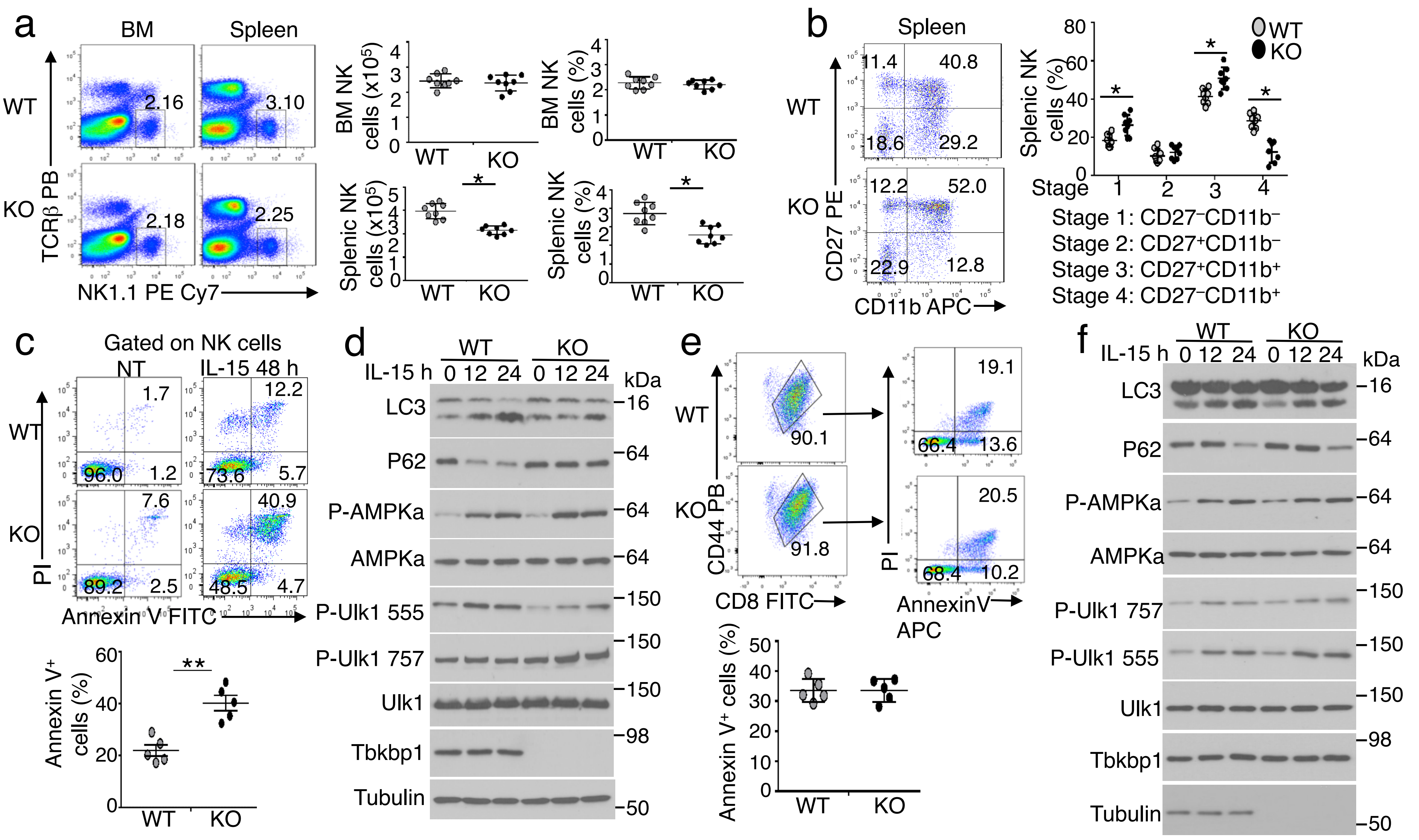

Supplementary Fig. 7. Tbkbp1 is required for IL-15-mediated survival and autophagy of NK, but not memory CD8^{+} T, cells. a,b, Flow cytometric analysis of NK cell frequency and absolute numbers (**a**) and maturation (**b**) in the bone marrow (BM) and spleen of WT or *Tbkbp1*-KO (KO) mice, presented as representative plots (left) or summary graphs (right). Four progressive maturation stages of spleen NK cells are indicated in **b**. **c**, Flow cytometric analysis of splenic NK cell apoptosis in WT or *Tbkbp1*-KO (KO) that were either not treated (NT) or incubated with IL-15 for 48 h. **d**, IB analysis of the indicated phosphorylated (P-) and total proteins in IL-15-stimulated WT or *Tbkbp1*-KO (KO) NK cells. **e,f**, Memory CD8^{+} T cells were generated by stimulating WT or *Tbkbp1*-KO (KO) naïve CD8^{+} T cells with anti-CD3 plus anti-CD28 in the presence of IL-2 for 3 days and then cultured in the presence with IL-15 for 3 additional days. The cells were starved overnight and restimulated with IL-15 for 24 h (**e**) or for the indicated time periods (**f**) and then subjected to flow cytometric (**e**) and IB (**f**) assays. Student's t-test **(a,b,c,e)**.

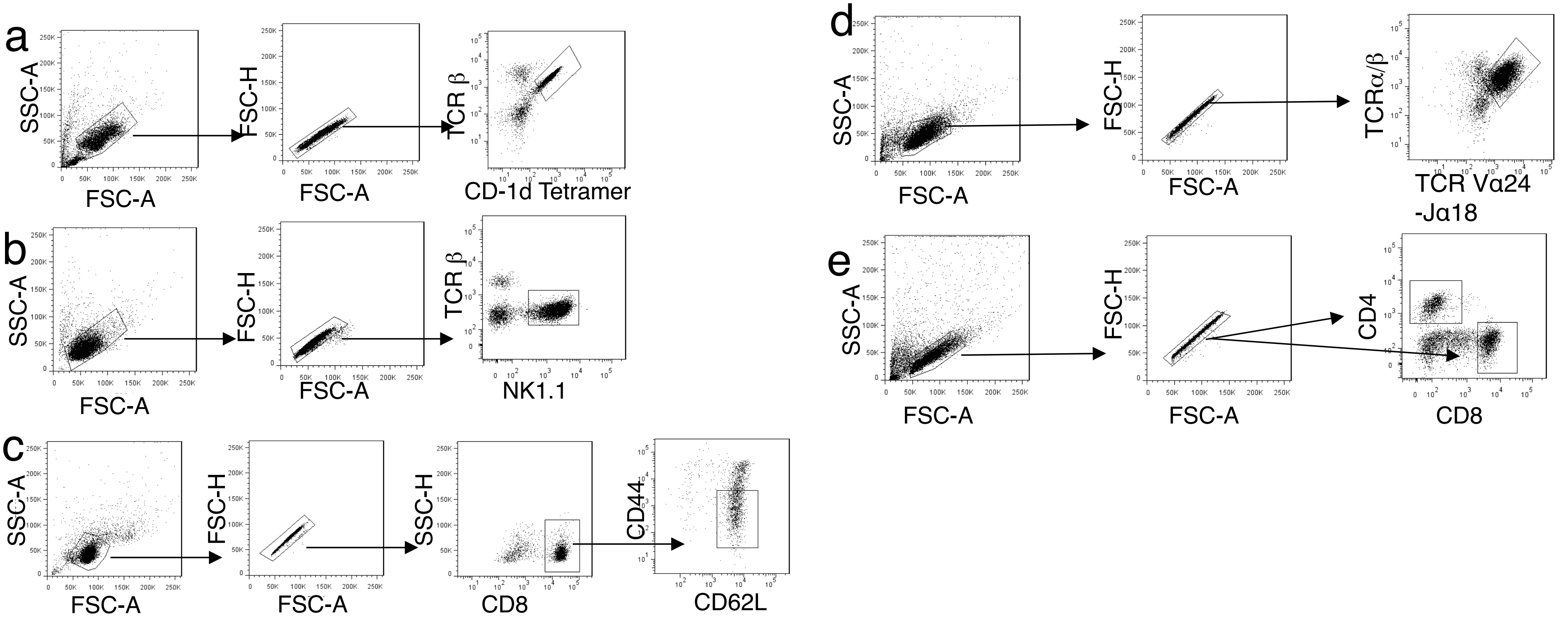

Supplementary Figure 8. Gating strategies used for cell sorting. a, Gating strategy to sort NKT cells from WT or Tbkbp1 KO mice (Fig. 1a, 1b, 4g, 5a, 6a, 6b,7a, and 7d). **b,** Gating strategy to sort NK cells from WT or Tbkbp1 KO mice (Fig. 1a,1b, and sFig 7d). (c)Gating strategy to sort naïve CD8 cells from WT or Tbkbp1 KO mice (sFig. 7e-f). **d,e,** Gating strategy to sort human CD4 cells, CD8 cells (d) or NKT cells (e) from *in vitro* PBMCs culture (sFig.1a-c).

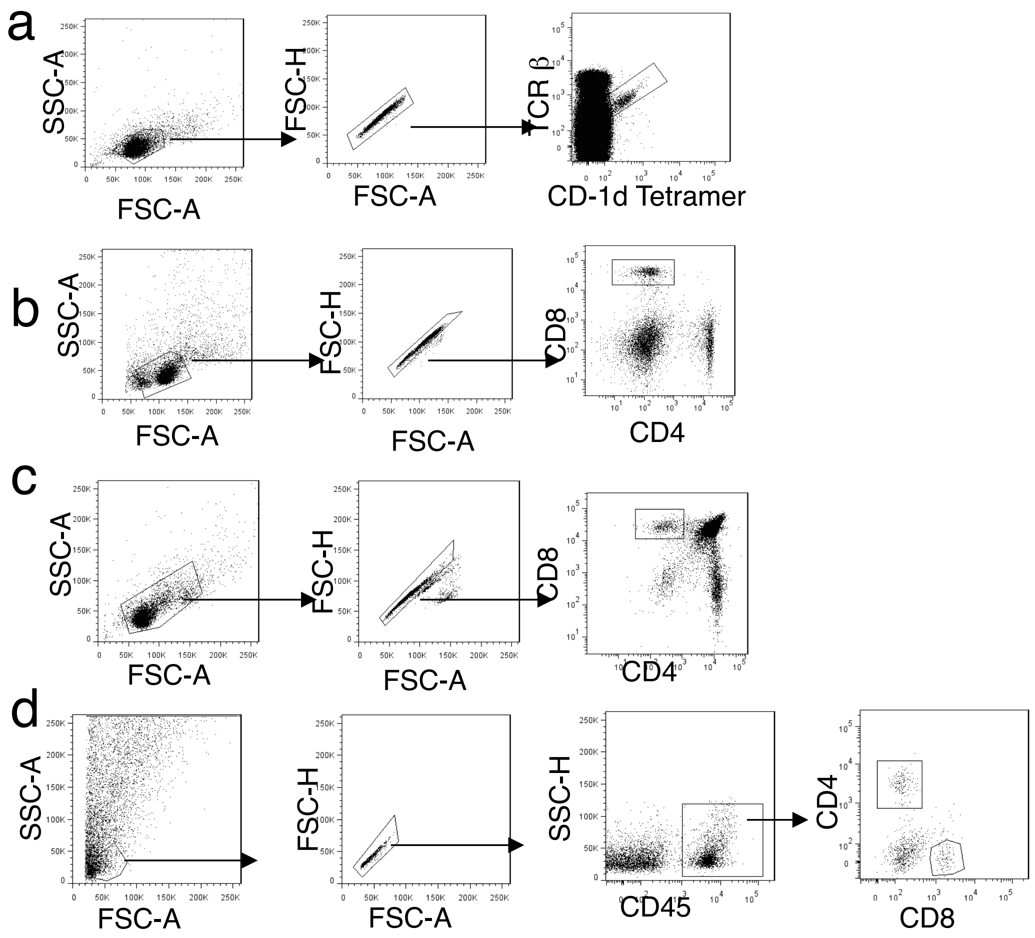

Supplementary Figure 9. Gating strategies used for flow cytometric analysis. a, Gating strategy for flow cytometric analysis of WT or Tbkbp1-KO (KO) NKT cells (Fig. 1c, 1d, 1f, 1g, 1h, 2a, 2c, 4a, 4c-f, 5b-e, 6f, sFig. 2b, 3d, 5a, 5c, 5e, 6c, 6h and 6i). **b,** Gating strategy to analyze splenic CD8 cells (Fig.3b and sFig.3b). **c,** Gating strategy to analyze thymic CD8 cells (Fig.3a, 3c, 3d and sFig.3a). **d,** Gating strategy for flow cytometric analysis of CD4 and CD8 T cells in tumors (Fig.3g, 3h and sFig 4b-e).

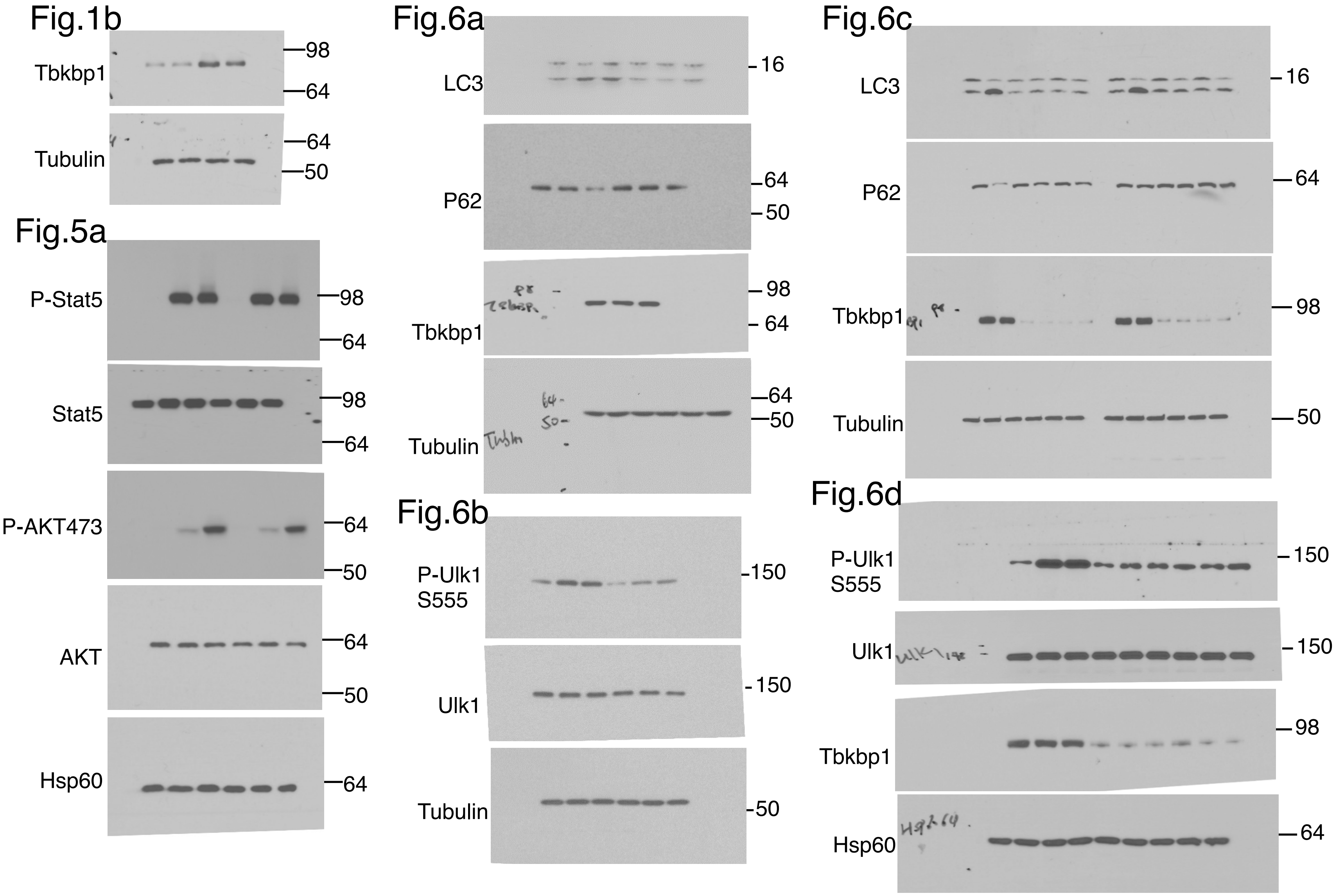

Supplementary Figure 10. Gel images for Figures 1b, 5a, and 6a-6d.

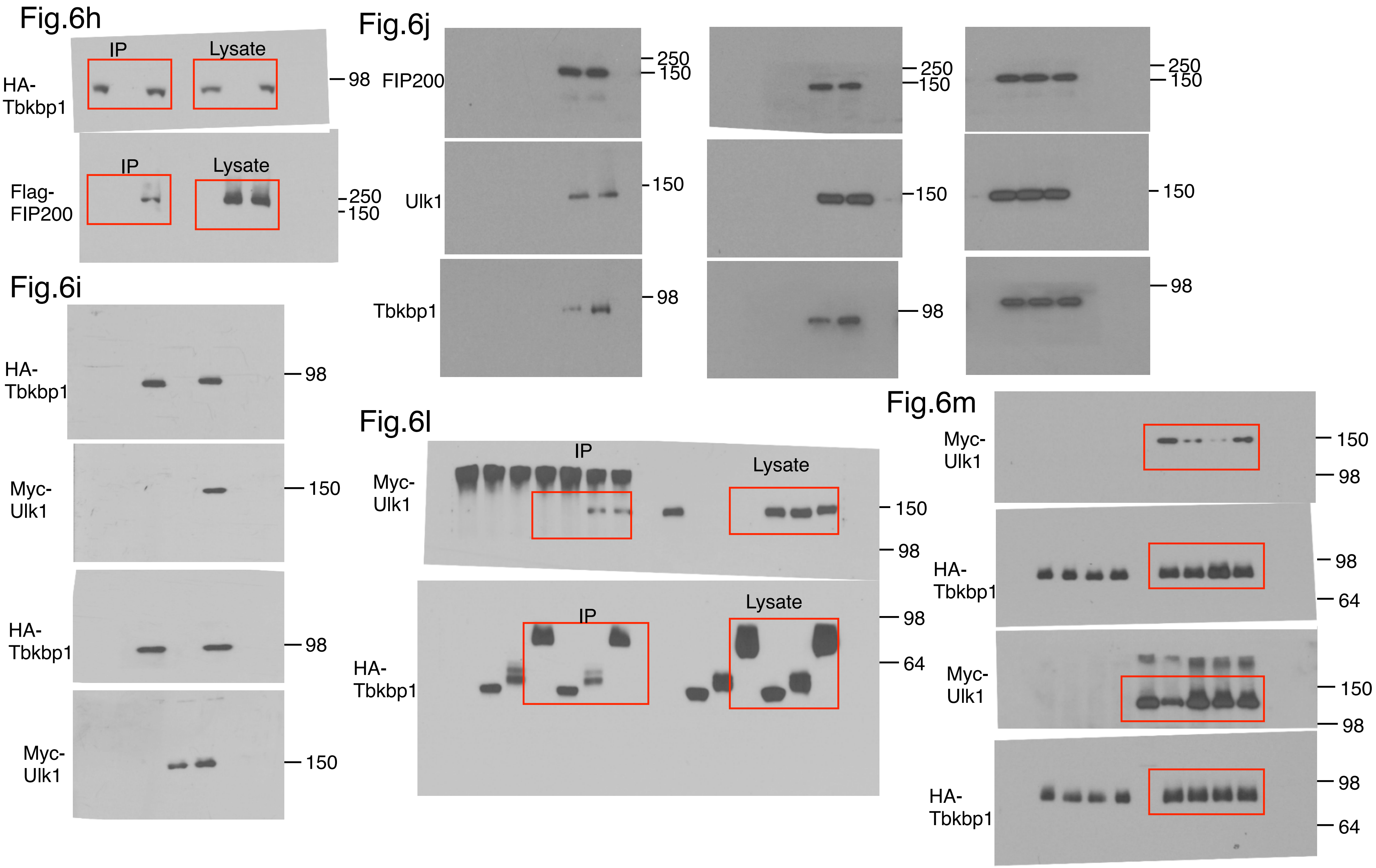

Supplementary Figure 11. Gel images for Figures 6h-6m

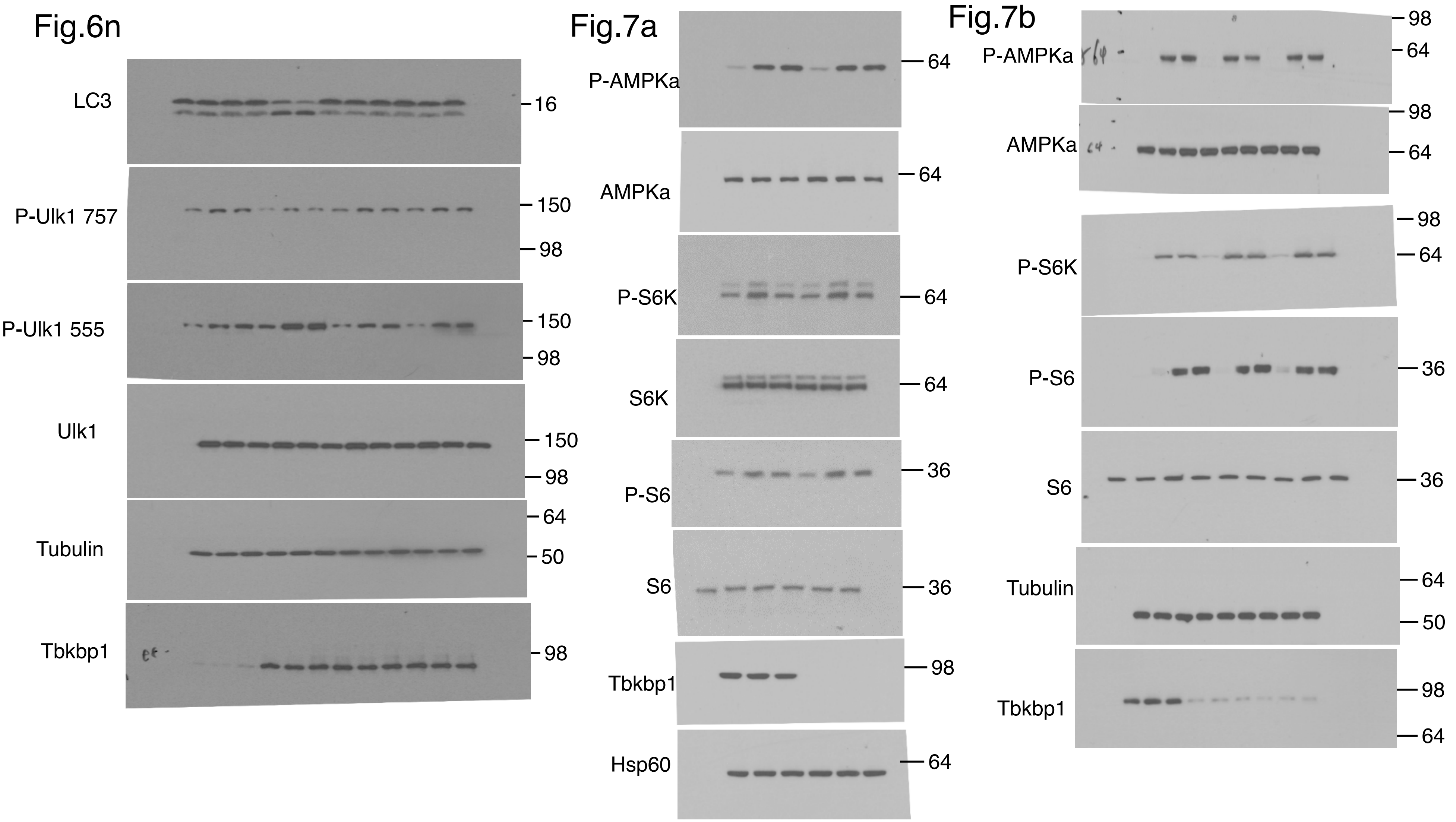

Supplementary Figure 12. Gel images for Figures 6n, 7a, and 7b

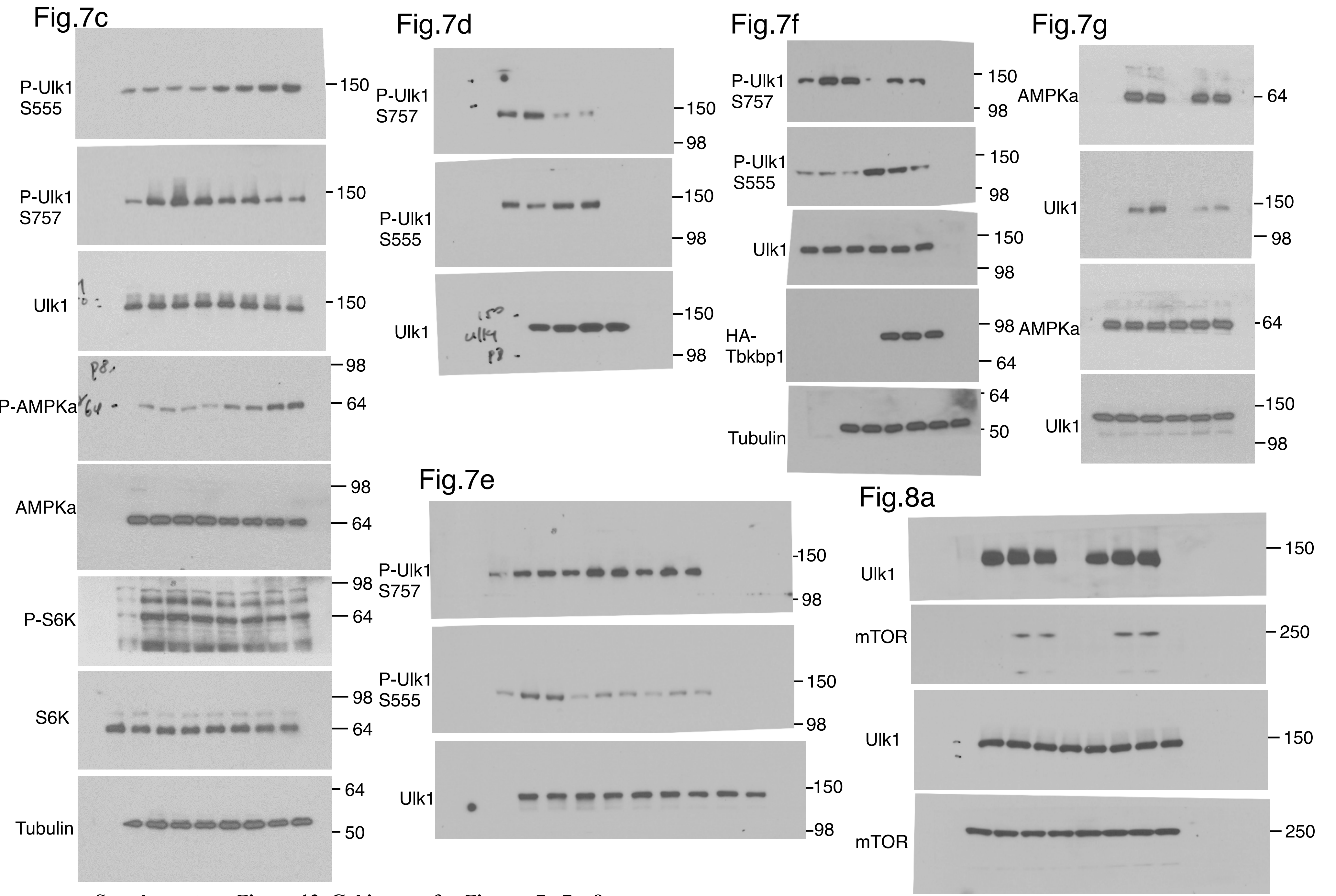

Supplementary Figure 13. Gel images for Figures 7c-7g, 8a

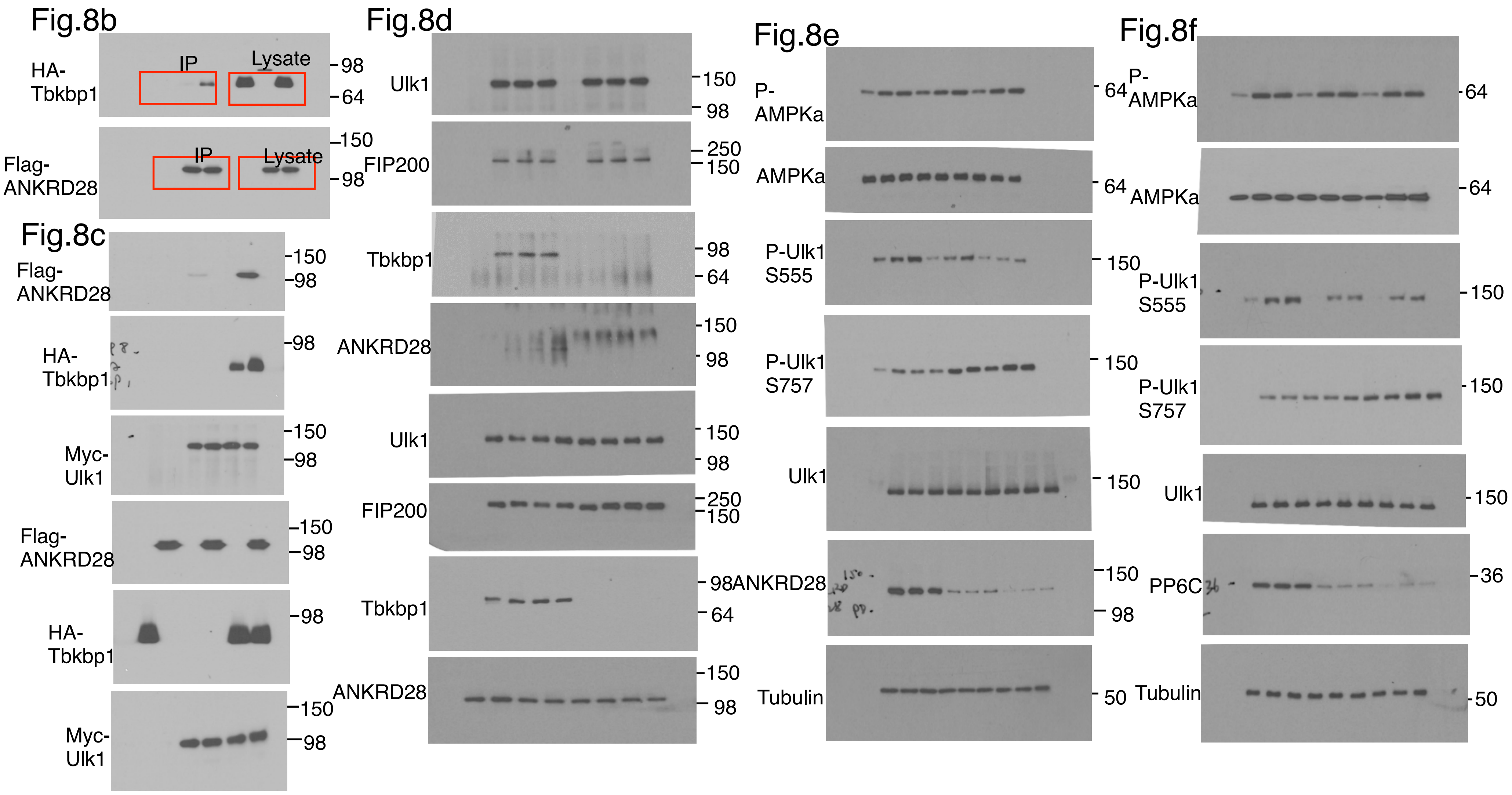

Supplementary Figure 14. Gel images for Figures 8b-8f

Supplementary Table 1 Gene-specific primers used in qRT-PCR experiments

Gene	Forward primer	Reverse primer
mTbkbp1	GGCTTGGGTCAAGAGAGTTG	GTTCCTGCAGCAAAGTCCTC
mBcl2	TCTGTGCACTGTGCATCTCTC	GACTTGGTGCATGGAACACTG
hTbkbp1	CTGATCAGTGACTTTGGAGAGG	CTCATGCTGGAACTGGTTGA
mActin	CGTGAAAAGATGACCCAGATCA	CACAGCCTGGATGGCTACGT
hActin	CGAGGCCCAGAGCAAGAGAG	CGGTTGGCCTTAGGGTTCAG
